# Supplementary material for: Engineering of a fluorescent chemogenetic reporter with tunable color for advanced live-cell imaging
Source: Nat Commun. 2021 Nov 30;12:6989. doi: 10.1038/s41467-021-27334-0 (PMC8633346; doi:10.1038/s41467-021-27334-0)
Supplement: Supplementary file 1 — Supplementary information [file 41467_2021_27334_MOESM1_ESM.pdf]

Supplementary Information for

Engineering of a fluorescent chemogenetic reporter with tunable color for advanced live-cell imaging

Hela Benaissa, Karim Ounoughi, Isabelle Aujard, Evelyne Fischer, Rosette Goïame, Julie Nguyen, Alison G. Tebo, Chenge Li, Thomas Le Saux, Giulia Bertolin, Marc Tramier, Lydia Danglot, Nicolas Pietrancosta, Xavier Morin, Ludovic Jullien & Arnaud Gautier*

* Arnaud Gautier
Email: arnaud.gautier@sorbonne-universite.fr

This PDF file includes:

Supplementary Notes 1- 2
Supplementary Fig. 1-24
Supplementary Tables 1-13
Supplementary References

Supplementary Note 1: Full description of the directed protein evolution experiments leading to oFAST, tFAST and pFAST

This supplementary note complements the main text, and details the full directed evolution experiments done during this study.

We used a combinatorial library of 10^6 variants of FAST generated by random mutagenesis and displayed on yeast cells (library A). Six different screenings in presence of either HBO-3M, HBO-3,5DM, HBT-3M, HBT-3,5DM, HBP-3M or HBP-3,5DM were performed by iterative rounds of fluorescence activated cell sorting (FACS) decreasing progressively chromophore concentrations through rounds (typically from 10 μM to 2.5 μM) in order to identify variants forming tighter and brighter assemblies (**Supplementary Fig. 2**,**3**). The screenings with HBO-3M, HBO-3,5DM and HBP-3,5DM showed an increase in cell population fluorescence indicating the selection of improved variants (**Supplementary Tables 5-7**). For each positive screening, we systematically isolated and sequenced twenty-four clones after the fifth, sixth and seventh round of FACS, and then further analyzed the performances of single clones by analytical flow cytometry. Five to ten individual clones with the highest fluorescence performances relative to FAST were expressed in bacteria and purified for in vitro characterization. The variants isolated from the three screenings formed tighter and brighter assemblies with the chromophore used for their selection (**Figure 2b,d**, **Supplementary Fig. 4a**, **Supplementary Tables 5-7**), in agreement with a successful molecular evolution.

By screening variants that combined mutations beneficial for the binding of HBO-3M (**Supplementary Table 5**) and HBO-3,5DM (**Supplementary Table 6**), we identified oFAST, an improved variant with the mutations Q41L, D71N, V83I, M109L and S117R, showing improved properties with both HBO-3M and HBO-3,5DM. oFAST binds HBO-3,5DM with a K_D of 3.0 μM, forming a blue fluorescent complex with 411/482 nm abs/em peaks and a fluorescent quantum yield ϕ = 30%, and it binds HBO-3M more tightly with a K_D of 0.73 μM, forming a blue fluorescent complex with 394/470 nm abs/em peaks and a fluorescent quantum yield ϕ = 11% (**Supplementary Table 2**).

The third positive selection allowed us to identify several variants able to bind HBP-3,5DM with 3 to 7 fold higher affinity, and giving complexes displaying higher fluorescence quantum yield. Beneficial mutations isolated from the three brighter variants were combined, leading eventually to three new variants binding HBP-3,5DM about 10-fold tighter than FAST and leading to higher fluorescent quantum yields (from ϕ = 22 to 26 %) (**Supplementary Table 7**). The five more promising variants were further characterized with the other members of the HBP series and with the chromophores of the HBT series. We discovered that the five variants were also able to form tighter and brighter assemblies with HBT-3,5DM (**Supplementary Table 8**), HBT-3,5DOM, HBP-3M and HBP-3,5DOM (**Supplementary Table 9**).

We thus used these five variant genes to construct a new library using DNA shuffling, which allows random DNA fragments recombination to access positive combinations. The five variant genes were randomly recombined to generate a library of 5.5×10^7 variants (library B), which was displayed on yeast cells. This new library was screened in presence of HBT-3,5DM, HBP-3,5DM and HBP-3,5DOM by iterative rounds of FACS as described above. The screening with HBP-3,5DOM did not allow us to identify variants with significantly better properties (**Supplementary Fig. 4b**, **Supplementary Table 9**). However, the screening with HBT-3,5DM allowed us to identify variants able to form brighter complexes (**Figure 2c**, **Supplementary Table 8**). The variant possessing the mutations G25R, Q41K, S72T, A84S, M95A, M109L and S117R relative to FAST, which we ultimately named tFAST, showed the most advantageous properties: it was shown to form a tight cyan fluorescent assembly with HBT-3,5DM (K_D = 0.33 μM, 435/497 nm abs/em peaks, ϕ = 11%) (**Supplementary Table 3**). On the other hand, the screening of the library B with HBP-3,5DM permitted us to identify an improved variant bearing the mutations K17N, G21E, G25R, A30V, Q41L, S72T, V83A, M95T, S117R, which was further refined by introduction of the mutation M109L found in several other improved variants. The resulting variant, named pFAST, binds HBP-3,5DM tightly with a K_D of 0.15 μM forming a bright green fluorescent assembly (ϕ = 27%, 465/520 nm abs/em peaks) (**Supplementary Table 4**, **Supplementary Table 7**).

Supplementary Note 2: Theoretical model for the determination of the thermodynamic dissociation constants of the dark chromophores

The thermodynamic dissociation constants (K_D) of the dark HBIR-3M and HBIR-3,5DM chromophores were determined by determining the apparent dissociation constant of HBP-3,5DM in presence of various concentrations of dark competitors. Considering that the fluorogen HBP-3,5DM and the protein interact to provide a fluorescent complex whereas the dark chromophores HBIR-3M or HBIR-3,5DM and the protein interact to form a non-fluorescent complex, we adopted the following three-state model

$$\begin{array}{ccc} F_1 + & R & \leftrightarrows F_1R \\ & + & \\ & F_2 & \\ & \uparrow\downarrow & \\ & F_2R & \end{array}$$

where F_1 denotes the fluorogen, F_2 denotes the dark chromophore, R denotes the protein and F_1R and F_2R denote the two possible complexes, characterized by the dissociation constants

$$K_{D,1} = \frac{[F_1]\ [R]}{[F_1R]}$$

and

$$K_{D,2} = \frac{[F_2]\ [R]}{[F_2R]}$$

where [X] is the concentration of the species X at equilibrium. At equilibrium, the fraction of protein R binding F_1 is given by

$$B = \frac{[F_1R]}{[R] + [F_1R] + [F_2R]} = \frac{\frac{[F_1]\ [R]}{K_{D,1}}}{[R] + \frac{[F_1]\ [R]}{K_{D,1}} + \frac{[F_2]\ [R]}{K_{D,2}}} = \frac{[F_1]}{[F_1] + K_{D,1}\left(1 + \frac{[F_2]}{K_{D,2}}\right)}$$
$$= \frac{[F_1]}{[F_1] + K_{D,app}}$$

with

$$K_{D,app} = K_{D,1}\left(1 + \frac{[F_2]}{K_{D,2}}\right)$$

By choosing $[F_1]_0$ and $[F_2]_0 >> [R]_0$, one can rewrite this equation assuming that $[F_1] \approx [F_1]_0$ and $[F_2] \approx [F_2]_0$. Knowing $K_{D,1}$, determination of the apparent thermodynamic dissociation constant $K_{D,app}$ at various $[F_2]_0$ enabled to determine $K_{D,2}$.

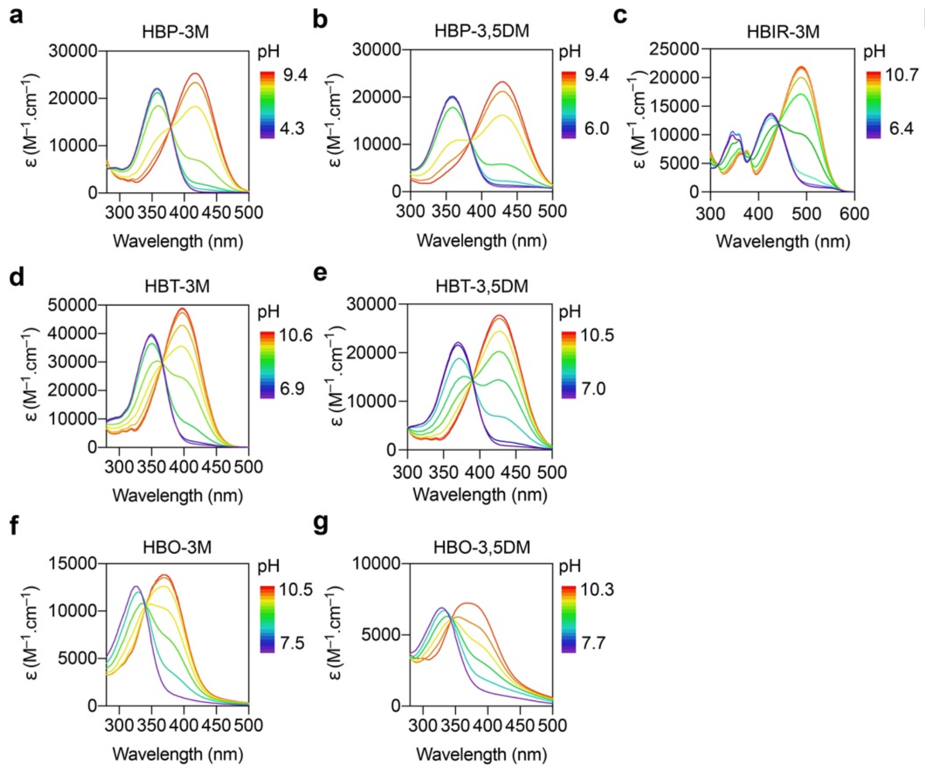

h

Fluorogens	pK_A
HBP-3M	8.15
HBP-3,5DM	8.25
HBT-3M	8.70
HBT-3,5DM	8.80
HBO-3M	8.90
HBO-3,5DM	9.05
HBIR-3M	8.29

Supplementary Fig. 1. Absorption spectra of fluorogenic chromophore at various pH. Absorption spectra of **a** HBP-3M, **b** HBP-3,5DM, **c** HBIR-3M, **d** HBT-3M, **e** HBT-3,5DM, **f** HBO-3M and **g** HBO-3,5DM in solution in function of pH. The spectra were recorded in 0.01 M Britton-Robinson buffer (1) (0.1 M ionic strength) at 25°C. **h** Extracted pK_A values. Source data for graphs are provided as a Source Data file.

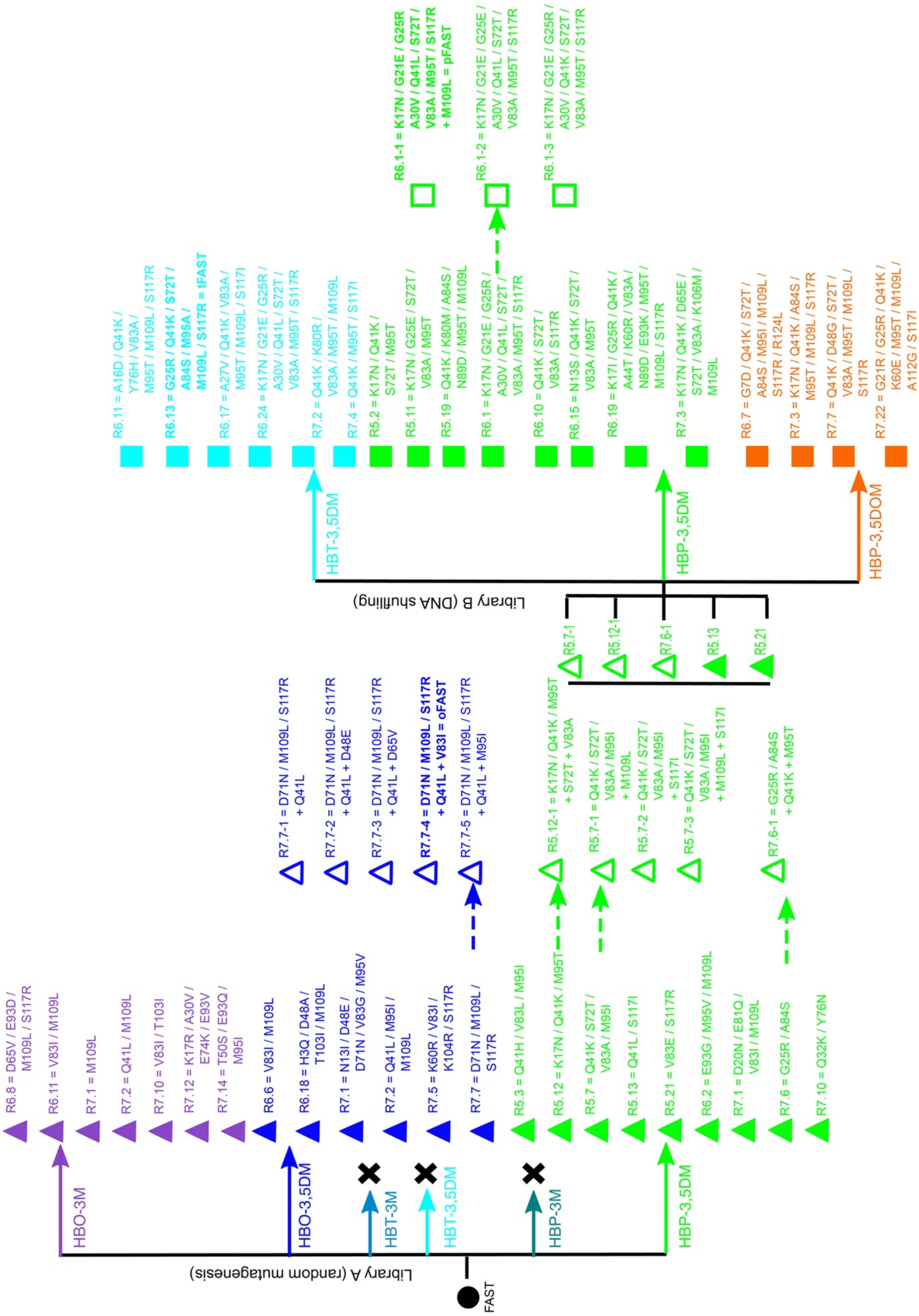

Supplementary Fig. 2. Evolutionary tree of the selections with HBO-3M (in purple), HBO-3,5DM (in blue), HBT-3,5DM (in cyan), HBP-3,5DM (in green) and HBP-3,5DOM (in orange). Selected clones were generated by directed evolution from initial libraries of mutants (solid arrows). The first library was constructed by random mutagenesis from the original FAST sequence (library A) and the second library was designed by DNA shuffling from five mutants, initially selected and rationally designed from the selection for HBP-3,5DM binders (library B). Additional mutations were introduced by rational design (dotted arrows). The crosses indicate unsuccessful selections. The mutations relative to FAST are indicated for each clone.

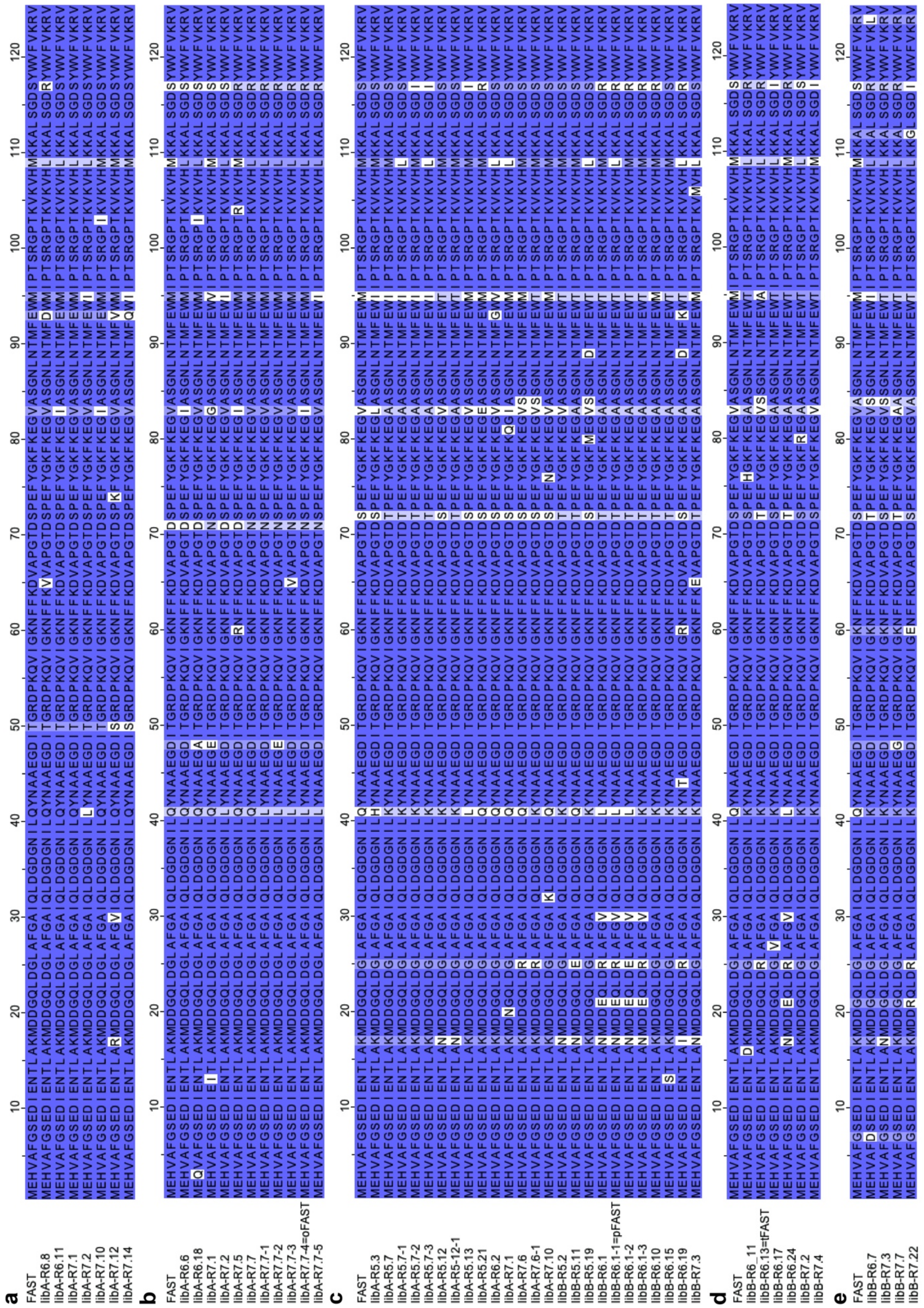

Supplementary Fig. 3. Sequence alignment of FAST and the clones identified from the selections with **a** HBO-3M, **b** HBO-3,5DM, **c** HBP-3,5DM, **d** HBT-3,5DM and **e** HBP-3,5DOM.

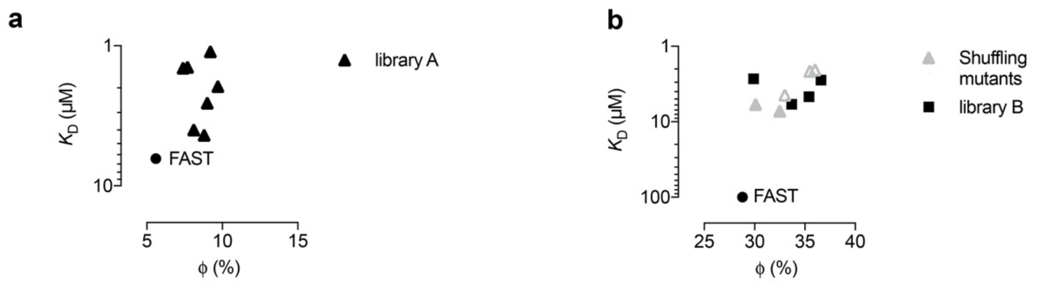

Supplementary Fig. 4. Characteristics of the selected clones. Thermodynamic dissociation constants (K_D) and fluorescent quantum yields (ϕ) of the clones isolated from the selections with **a** HBO-3M and **b** HBP-3,5DOM. Values are also given for FAST for comparison. **a** Mutants of HBO-3M selection were isolated from the library A (black triangles). **b** Mutants of HBP-3,5DOM were isolated from the library B (black squares) and compared to the shuffling mutants used to design the library B (gray triangles). Source data for graphs are provided as a Source Data file.

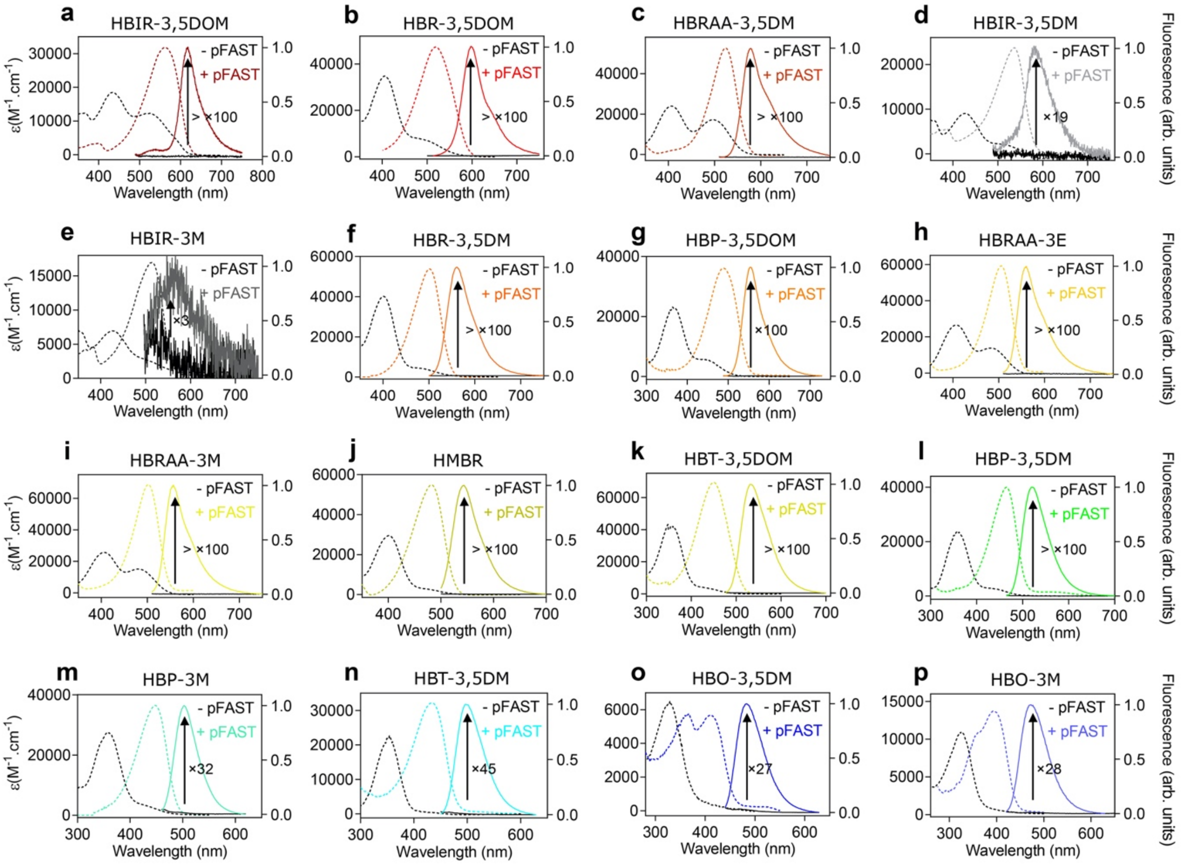

Supplementary Fig. 5. **Absorption and emission properties.** Absorption (dashed lines) and fluorescence (solid lines) spectra of the fluorogenic chromophores when free in solution (dark line) and bound to pFAST (colored lines) for **a** HBIR-3,5DOM, **b** HBR-3,5DOM, **c** HBRAA-3,5DM, **d** HBIR-3,5DM, **e** HBIR-3M, **f** HBR-3,5DM, **g** HBP-3,5DOM, **h** HBRAA-3E, **i** HBRAA-3M, **j** HMBR, **k** HBT-3,5DOM, **l** HBP-3,5DM, **m** HBP-3M, **n** HBT-3,5DM, **o** HBO-3,5DM and **p** HBO-3M. Spectra were recorded using chromophore concentrations ranging from 3 – 15 μM and pFAST at 40 μM in pH 7.4 phosphate buffer saline at 25°C. Fluorescence enhancement upon binding are indicated on the graphs. Source data for graphs are provided as a Source Data file.

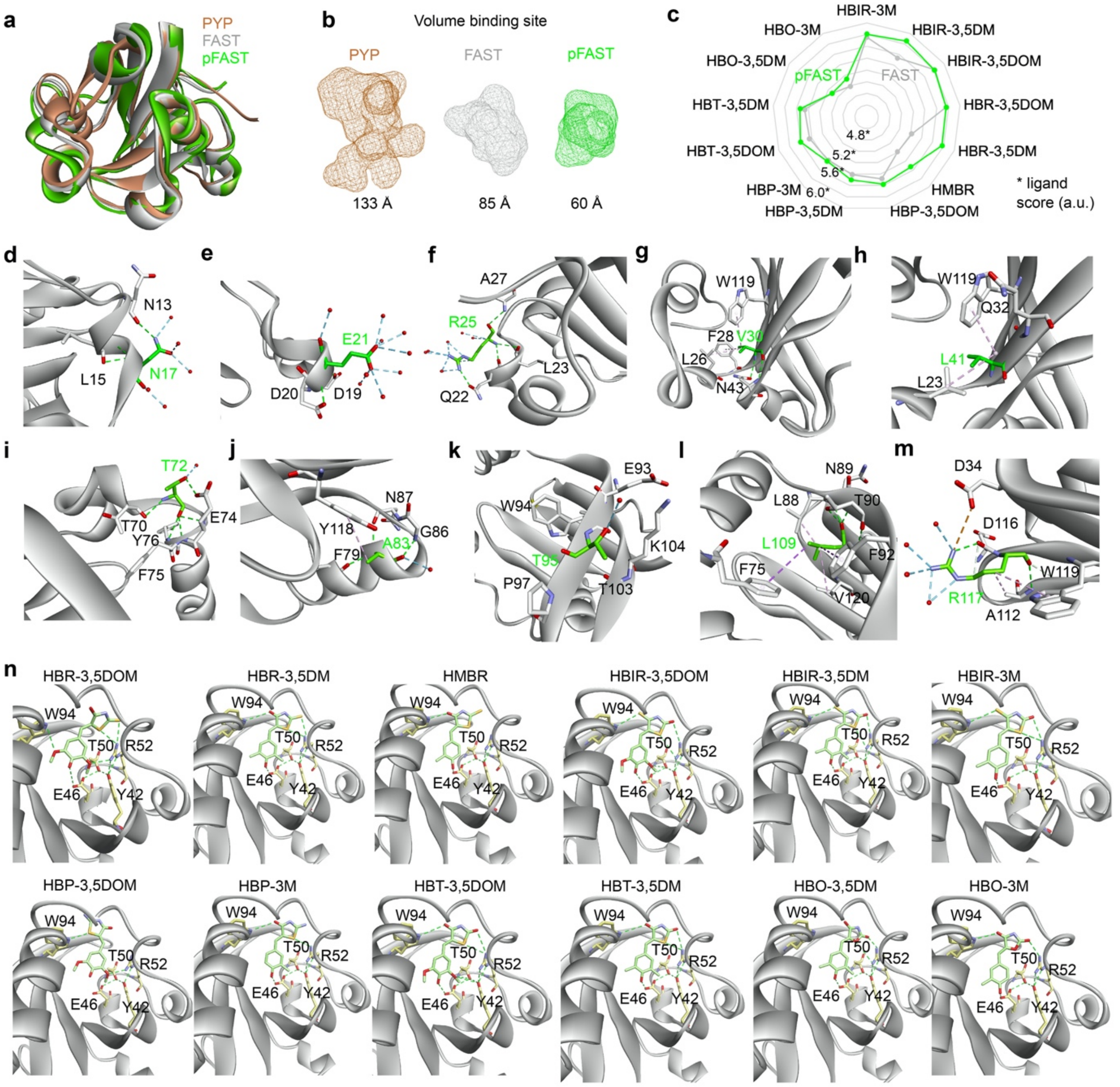

Supplementary Fig. 6. Structural homology models. a Structural models of FAST and pFAST generated from the tridimensional crystal structure of the *Halorhodospira halophila* Photoactive Yellow Protein PYP (PDB: 6P4I). **b** Volumes of the ligand binding sites of PYP, FAST and pFAST. **c** In silico binding affinities (ligscore 2) of FAST and pFAST for the HBIR, HBR, HBR, HBT and HBO series. **d-m** Mapping of the residues involved in polar (green dashed lines) and apolar interactions (purple dashed lines) with the residues found mutated in pFAST. **n** Molecular docking of pFAST-chromophore assemblies.

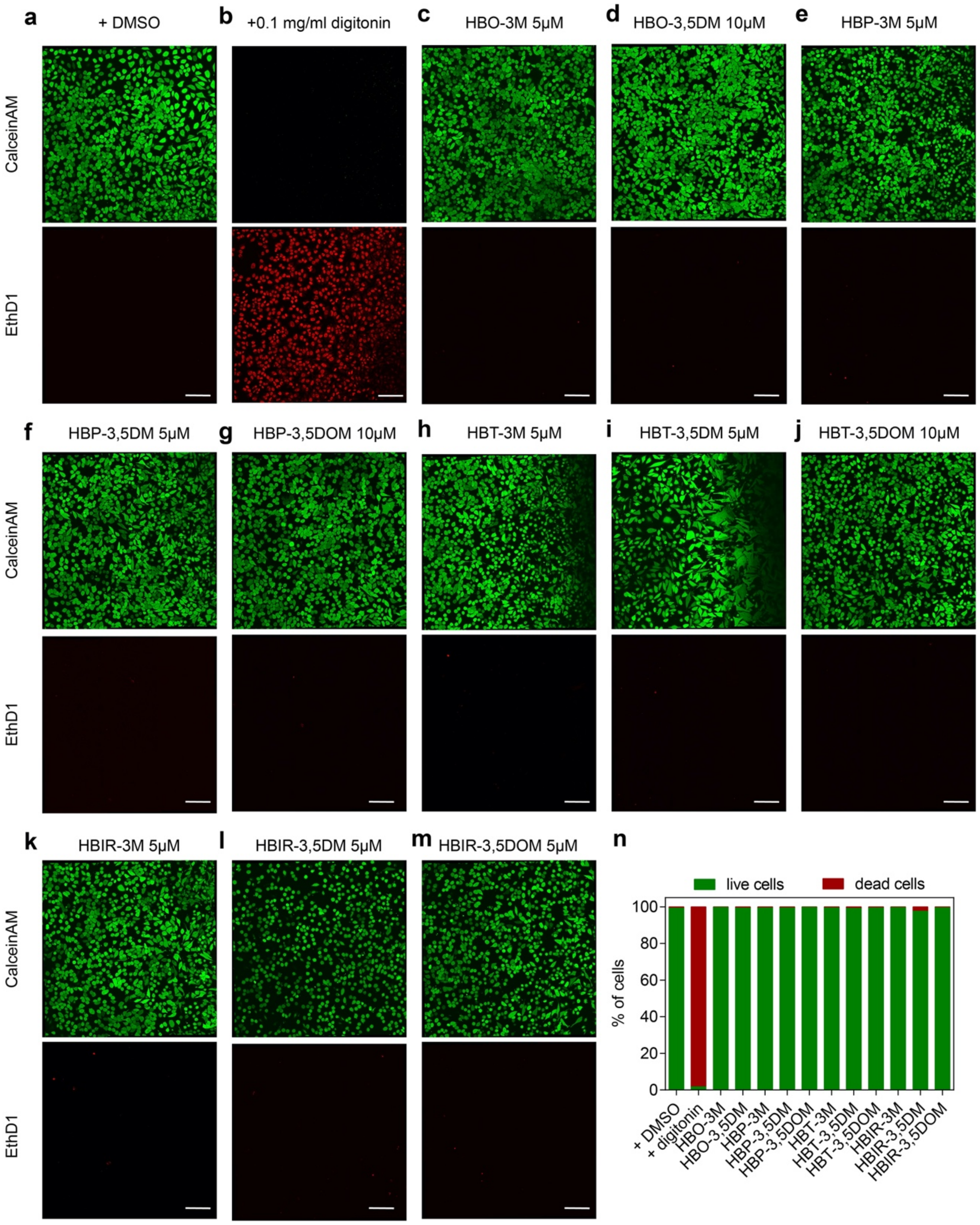

Supplementary Fig. 7. Two-color fluorescence viability assay. HeLa cells were incubated for 24 h with solutions of **a-b** 0.1 % of DMSO, **c** HBO-3M at 5 μM, **d** HBO-3,5DM at 10 μM, **e** HBP-3M at 5 μM, **f** HBP-3,5DM at 5 μM, **g** HBP-3,5DOM at 10 μM, **h** HBT-3M at 5 μM, **i** HBT-3,5DM at 5 μM, **j** HBT-3,5DOM at 5 μM, **k** HBIR-3M at 5 μM, **l** HBIR-3,5DM at 5 μM and **m** HBIR-3,5DOM at 5 μM. Control experiments of HeLa cells non-incubated with dye (**a**, live cells) or incubated for 30 min with 0.1 mg/ml digitonin (**b**, dead cells) are shown. Cell viability was tested with calceinAM and EthD1 probes (LIVE/DEAD® viability/cytotoxicity assay kit). CalceinAM is a cell-permeant profluorophore cleaved by intracellular esterases releasing a uniform green fluorescence polyanionic calcein in live cells (green channel). EthD1 (Ethidium homodimer-1) is a non-permeant nucleic acid fluorescent stain that enters only cells with damaged membranes and undergoes a fluorescence enhancement upon binding to nucleic acids, thereby producing a bright red fluorescence in dead cells (red channel). Cell fluorescence was evaluated by confocal microscopy. Identical imaging settings were used for the different experiments. **n** Quantification of the proportion of live (green) and dead (red) cells. Scale bars, 100 μm. Source data for graphs are provided as a Source Data file.

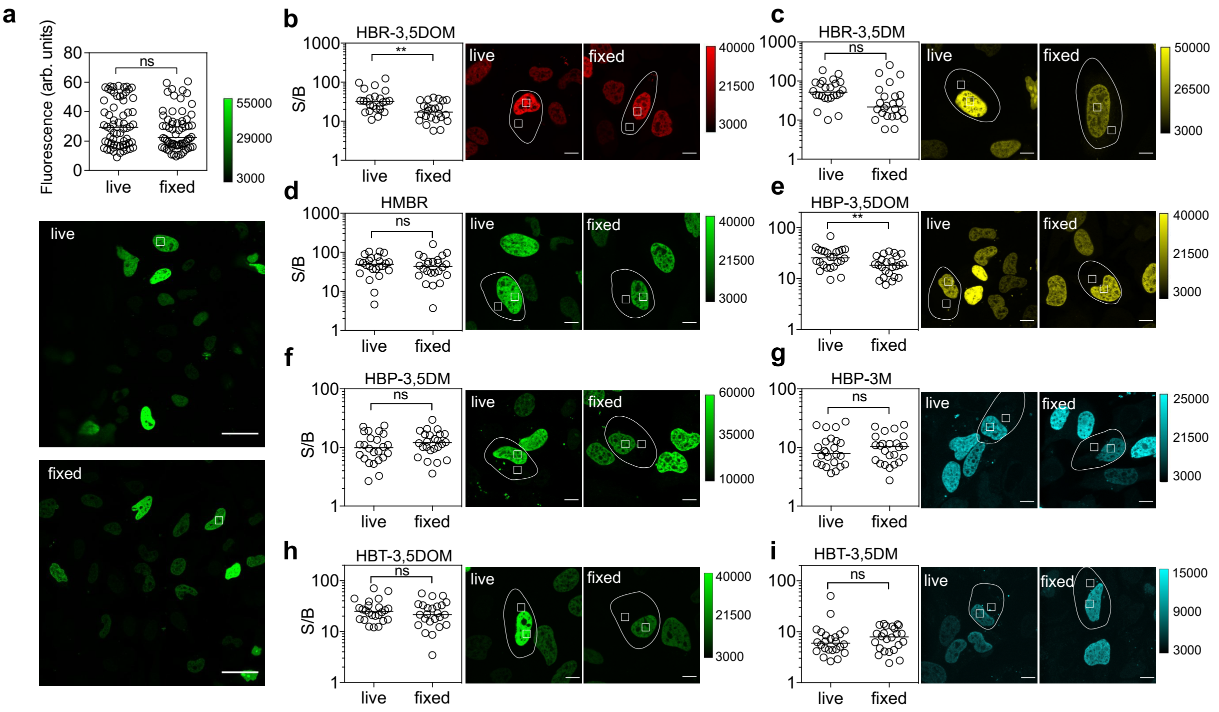

Supplementary Fig. 8. Imaging of H2B-pFAST in live and fixed HeLa cells after addition of various fluorogenic chromophores. a Nuclear fluorescence in live and fixed cells incubated with 5 μM of HMBR and imaged with identical microscope settings (n = 64 cells, 2 experiments). Median values are reported and a Wilcoxon test was conducted to compare live and fixed cell populations (ns = not significative). Scale bars, 40 μm. **b-i** Comparison of signal (nucleus) to background (cytosol) (S/B) ratio between live and fixed cells incubated with **b** HBR-3,5DOM at 5 μM, **c** HBR-3,5DM at 5 μM, **d** HMBR at 5 μM, **e** HBP-3,5DOM at 10 μM, **f** HBP-3,5DM at 5 μM, **g** HBP-3M at 5 μM, **h** HBT-3,5DOM at 5 μM and **i** HBT-3,5DM at 5 μM. For each experiment, live and fixed cells were imaged with identical microscope settings (n = 24 cells, 2 experiments). Median of S/B ratios are reported and a two-sided Wilcoxon test was conducted to compare live and fixed cell populations (ns = not significant, ** $p < 0.01$ [panel **b** $p = 0.008$; panel **g** $p = 0.005$]). (see **Supplementary Table 13** for imaging settings). Scale bars, 10 μm. Source data for graphs are provided as a Source Data file.

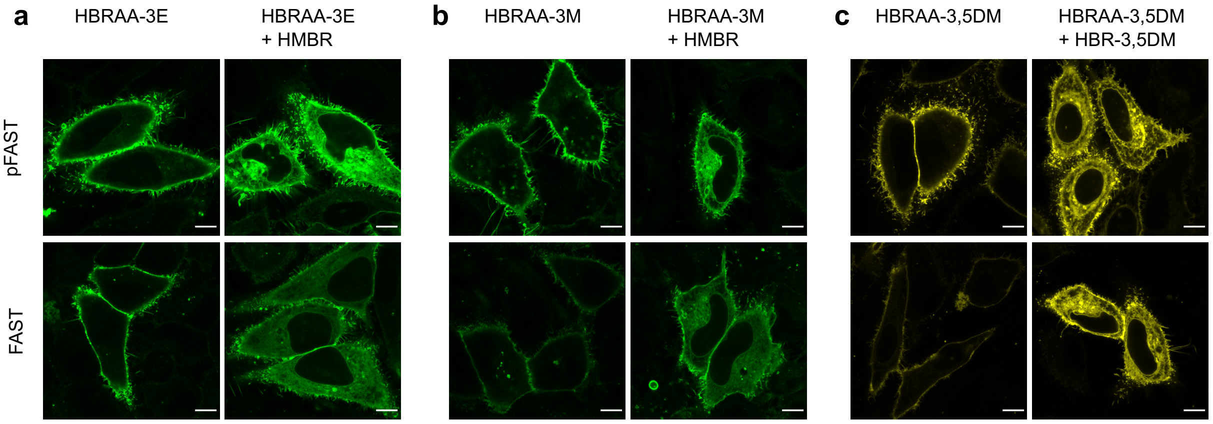

Supplementary Fig. 9. Selective imaging of cell-surface proteins. Confocal micrographs of HeLa cells expressing a secreted transmembrane domain fused to pFAST versus FAST labeled with impermeant fluorogens **a** HBRAA-3E at 10 μM, **b** HBRAA-3M at 10 μM and **c** HBRAA-3,5DM at 10 μM. Subsequent addition of 5 μM of membrane-permeant **a,b** HMBR or **c** HBR-3,5DM revealed the total pool of proteins expressed at the surface and within the secretory pathway. For each experiment, FAST and pFAST were imaged under the same imaging settings. The detection settings were adjusted to take into account the difference of brightness of impermeant and membrane-permeant fluorogens for each experiment. (see **Supplementary Table 13** for imaging settings). Experiments were repeated 4 times with similar results. Scale bars, 10 μm.

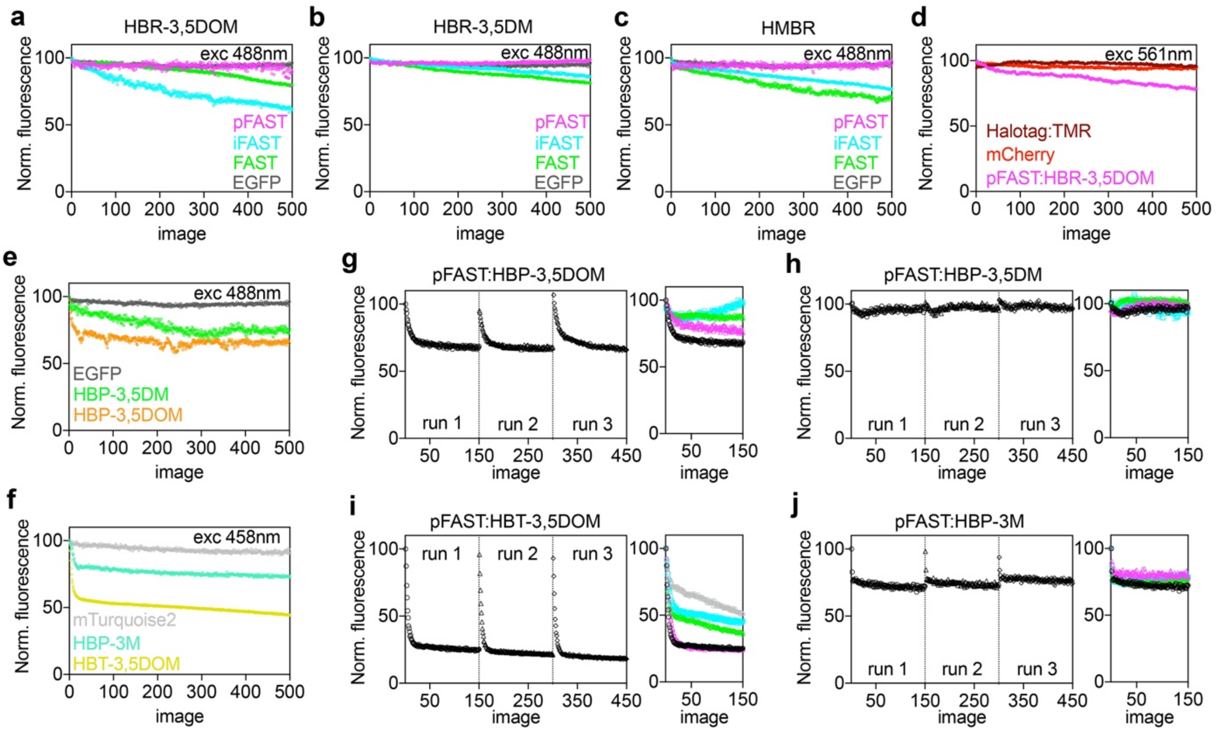

Supplementary Fig. 10. **In-cell photostability of pFAST. a-c** Photostability of pFAST compared to FAST and iFAST in presence of **a** 10 µM HBR-3,5DOM, **b** 5 µM HBR-3,5DM and **c** 5 µM HMBR. Photostability of EGFP is also given for comparison. Cells were illuminated with a 488 nm laser excitation (with a power of 4.4 kW/cm^2 at the specimen plane) and 500 images were acquired every 2 s, n = 3 cells per curve. **d** Comparison of the photostability of pFAST:HBR3,5DOM, mCherry and HaloTag labeled with tetramethyl rhodamine (TMR). Cells were illuminated with a 561 nm laser excitation (with a power of 8.7 kW/cm^2 at the specimen plane) and 500 images were acquired every 2 s, n = 3 cells per curve. **e** Photostability of pFAST in presence of 10 µM HBP-3,5DOM and 5 µM HBP-3,5DM under 488 nm laser excitation (with a power of 4.4 kW/cm^2 at the specimen plane). Photostability of EGFP is also given for comparison. 500 images were acquired every 2 s, n = 3 cells per curve. **f** Photostability of pFAST in presence of 5 µM HBT-3,5DOM and 5 µM HBP-3M under continuous 458 nm laser excitation (with a power of 3.5 kW/cm^2 at the specimen plane). Photostability of mTurquoise2 is also given for comparison. 500 images were acquired every 2 s, n = 3 cells per curve. **g-h** Photostability of pFAST in presence of **g** 10 µM HBP-3,5DOM and **h** 5 µM HBP-3,5DM under 488 nm laser excitation with a power of 4.7 kW/cm^2 (black and green curves) and 2.4 kW/cm^2 (pink and cyan curves) at the specimen plan. 150 images were acquired every 2s (black and pink curves) and 5s (green and cyan curves) followed by 60s in the dark before acquisition was restarted. **i-j** Photostability of pFAST in presence of **i** 5 µM HBT-3,5DOM and **j** 5 µM HBP-3M under 458 nm laser excitation with a power of 3.7 kW/cm^2 (black and green curves) and 2.4 kW/cm^2 (pink, cyan and gray curves) at the specimen plan. 150 images were acquired every 2 s (black and pink curves), every 5 s (green and cyan curves) and every 10 s (gray curve only for HBT-3,5DOM) followed by 60 s in the dark before acquisition was restarted. **a-j** The proteins were expressed in **a-c**,**e-j** the cytoplasm in HeLa cells or **d** as fusion to nuclear H2B and images were acquired using a scanning confocal microscope with a pixel dwell time of 2.55 µs (see **Supplementary Table 13** for imaging settings). Source data for graphs are provided as a Source Data file.

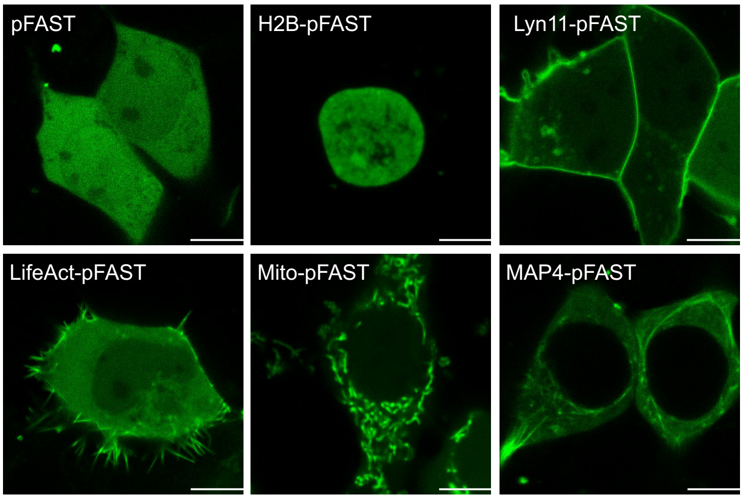

Supplementary Fig. 11. Selective imaging of pFAST in live mammalian cells. Confocal micrographs of live HEK293T cells expressing pFAST fused to: histone H2B, lyn11 (inner membrane-targeting motif), LifeAct (actin binding peptide domain), mito (mitochondrial targeting motif) and to microtubule-associated protein (MAP) 4 and labeled with 5 μM HBP-3,5DM (see **Supplementary Table 13** for imaging settings). Experiments were repeated > 3 times with similar results. Scale bars, 10 μm.

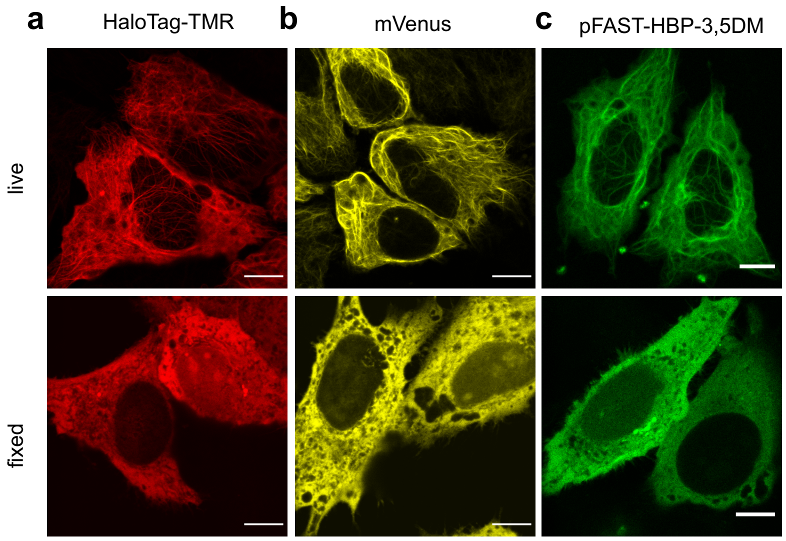

Supplementary Fig. 12. Confocal micrographs of live and fixed HeLa cells expressing MAP4 fused to **a** HaloTag labeled with 2.5 μM of HaloTag® TMR Ligand, **b** mVenus and **c** pFAST labeled with HBP-3,5DM at 5 μM (see **Supplementary Table 13** for imaging settings). Experiments were repeated 3 times with similar results. Scale bars, 10 μm.

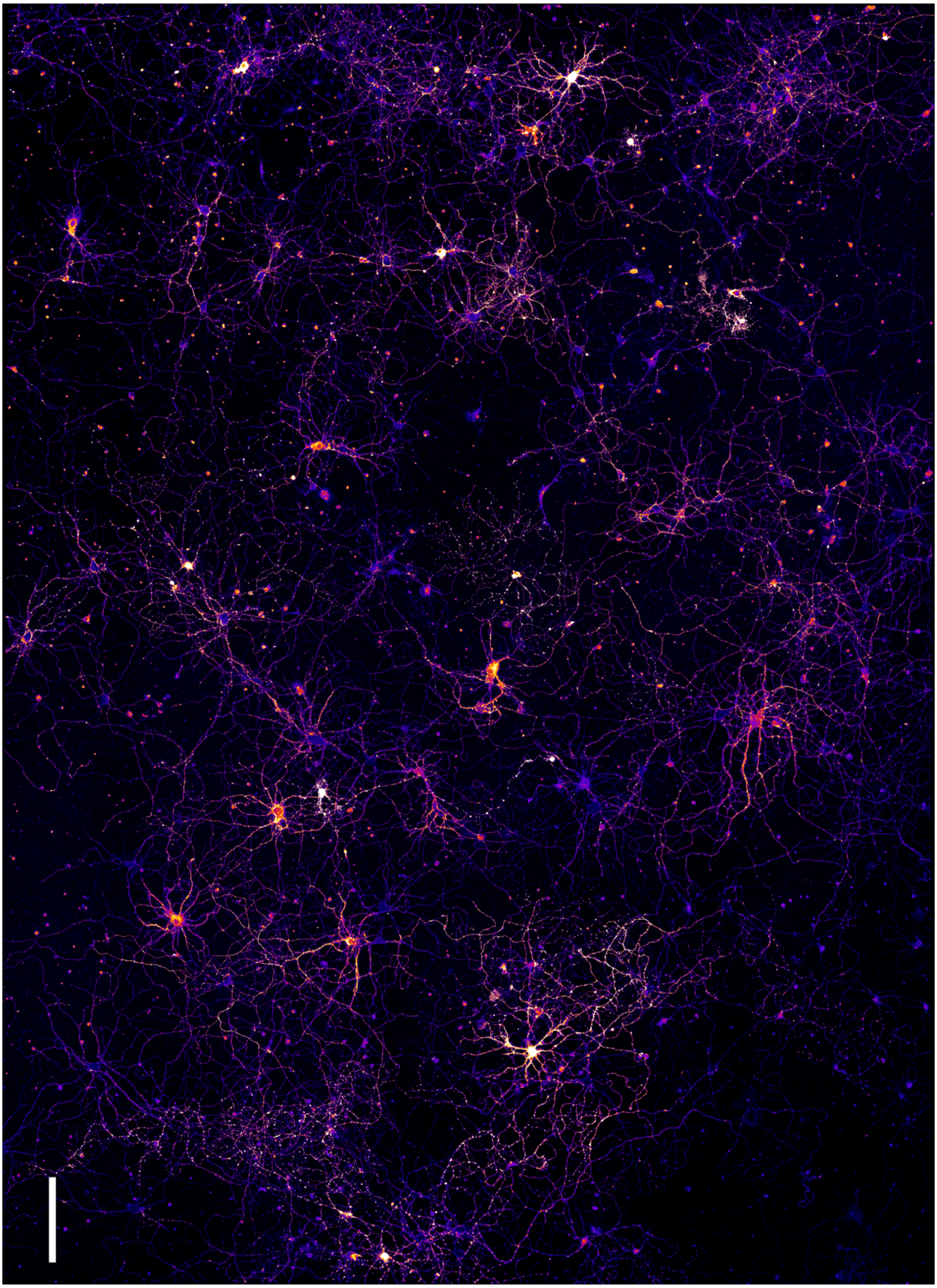

Supplementary Fig. 13. Selective imaging of hippocampal neuronal network. Tiles of confocal micrographs of dissociated hippocampal neurons transfected with a plasmid encoding pFAST fused to lyn11 (inner membrane targeting motif), and labeled with 10 μM HBR-3,5DOM (see **Supplementary Table 13** for imaging settings). Experiment was repeated 12 times with similar results. The huge field of view enables to illustrate the high transfection rate and viability of transfected fragile cells like neurons. Artificial look up table 'fire' color is used to illustrate intensity dynamics. Scale bar, 200 μm.

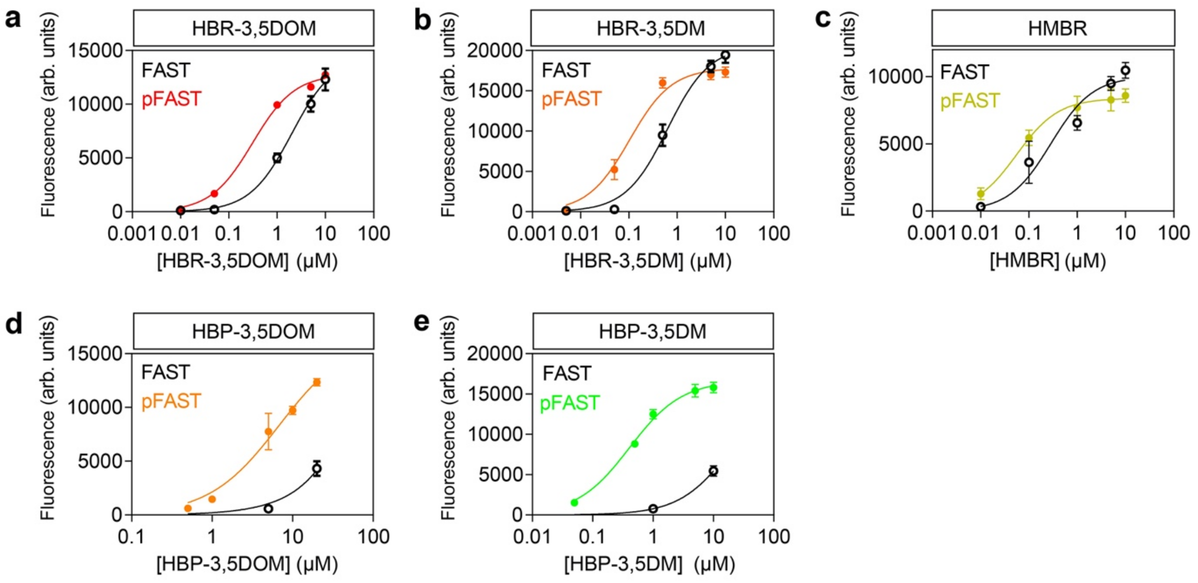

Supplementary Fig. 14. **Labeling efficiency in live cells.** Fluorescence of HEK 293T cells expressing cytoplasmic pFAST (colored lines) versus FAST (black lines) in the presence of increased concentrations of **a** HBR-3,5DOM, **b** HBR-3,5DM, **c** HMBR, **d** HBP-3,5DOM and **e** HBP-3,5DM (n = 3 replicates). Fluorescence was measured by flow cytometry. Data are presented as mean values ± SD of 3 experiments. Source data for graphs are provided as a Source Data file.

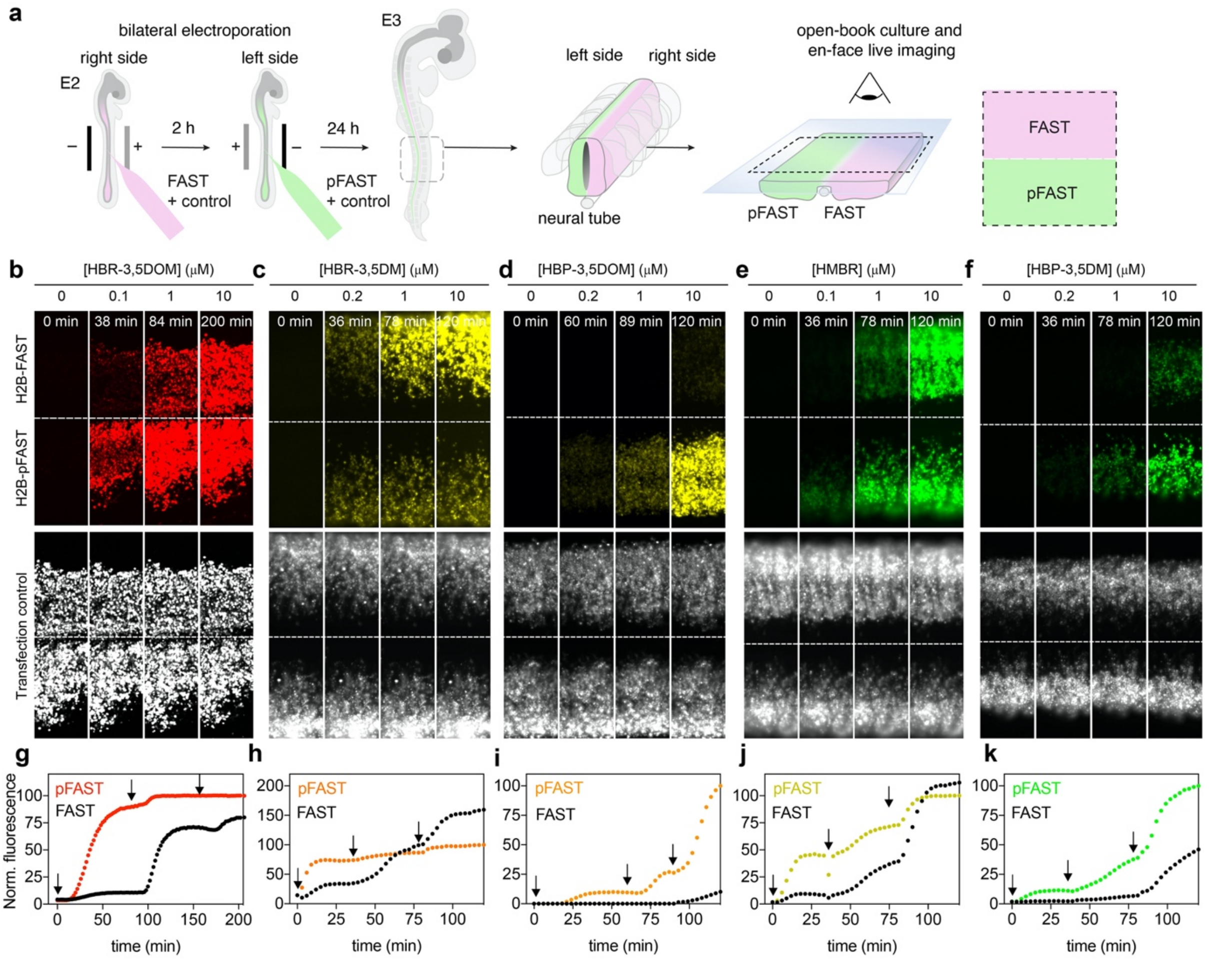

Supplementary Fig. 15. **Labeling efficiency in chicken embryos. a-f** Plasmids encoding H2B-pFAST and H2B-FAST were electroporated in each side of the neural tube in ovo at embryonic day 2 (E2, HH stage 13-14). **b** EGFP or **c-f** mCherry reporters were co-injected with each construct as a transfection efficiency control. 24 h later, embryos with homogeneous bilateral reporter expression in the neural tube were dissected and imaged. Time-lapse imaging upon sequential addition of fluorogenic chromophore were acquired using fluorescence microscopy. Conditions: **b** 0.1, 1 and 10 μM HBR-3,5DOM (see also **Supplementary Movie 1)** ; **c** 0.2, 1 and 10 μM HBR-3,5DM ; **d** 0.2, 1 and 10 μM of HBP-3,5DOM ; **e** 0.1, 1 and 10 μM of HMBR and **f** 0.2, 1 and 10 μM of HBP-3,5DM (see **Supplementary Table 13** for imaging settings). Experiments were repeated 3 times with similar results. **g-k** Fluorescence intensities of pFAST and FAST were analyzed over time and normalized by the maximal fluorescence value of pFAST for each fluorogenic chromophore. The sequential addition of the fluorogenic chromophore at the different concentrations are indicated by arrows. Source data for graphs are provided as a Source Data file.

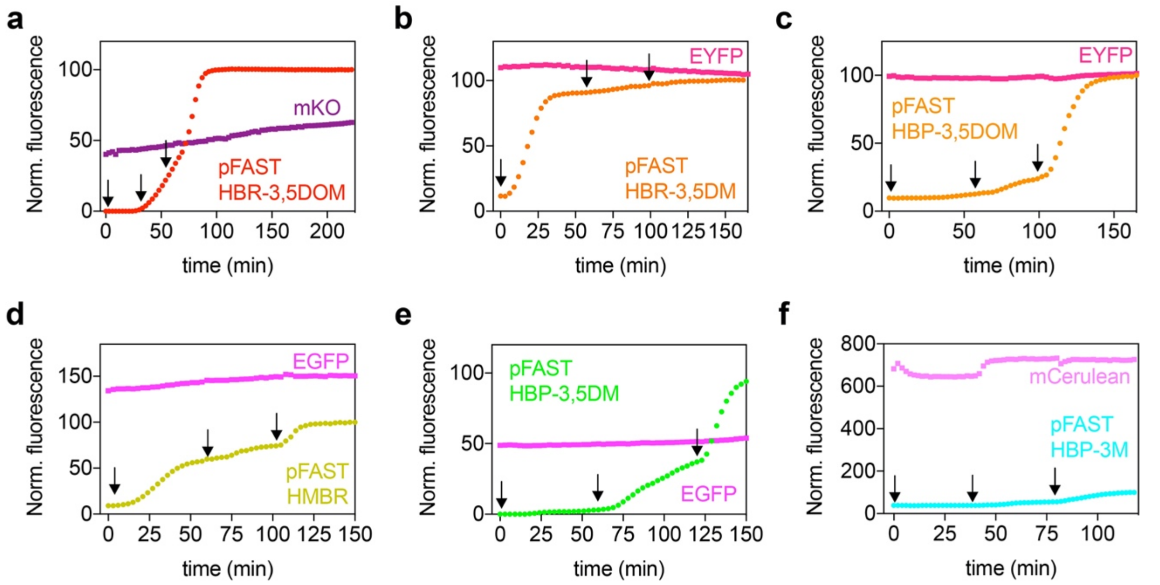

Supplementary Fig. 16. Comparison of pFAST with various fluorescent proteins in chicken embryos. Embryos expressing pFAST and the fluorescent proteins **a** mKO, **b,c** EYFP, **d,e** EGFP and **f** mCerulean in each side of the neural tube were dissected and labeled by sequential additions of various concentrations of fluorogenic chromophores (arrows indicate additions). **a** 0.1, 1, 10 μM of HBR-3,5DOM; **b** 0.2, 1, 10 μM of HBR-3,5DM; **c** 0.2, 1, 10 μM of HBP-3,5DOM; **d** 0.2, 1, 10 μM of HMBR; **e** 0.2, 1, 10 μM of HBP-3,5DM and **f** 0.2, 1, 10 μM of HBP-3M. Time-lapse fluorescence imaging allowed to monitor the temporal evolution of the fluorescence intensity (**Figure 5** in main text shows the initial and final images). Fluorescence intensities were normalized by the maximal fluorescence value of pFAST for each experiment. Source data for graphs are provided as a Source Data file.

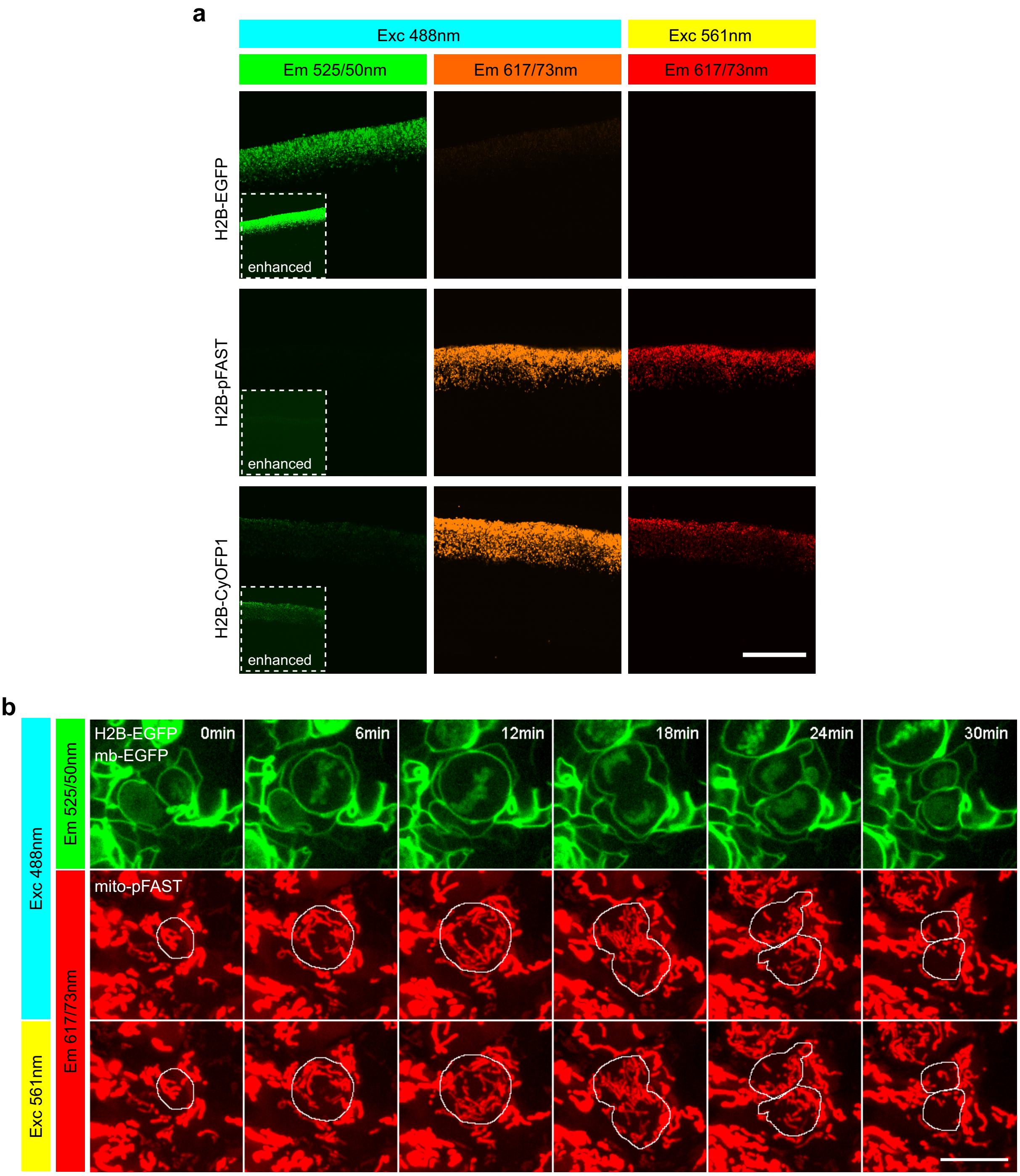

Supplementary Fig. 17. Two-color imaging with single excitation in chicken embryo. **a** Plasmids encoding H2B-EGFP, H2B-pFAST and H2B-CyOFP1 were electroporated on one side of neural tube in ovo at embryonic day 2 (E2, HH stage 13-14). 24 h later, embryos were dissected and imaged by spinning disk confocal microscopy with the indicated imaging settings. pFAST was labeled with HBR-3,5DOM prior to imaging. The experiment was done once. Scale bar, 300 μm**. b** Plasmids encoding H2B-EGFP, mb-EGFP (membrane-targeted EGFP), and mito-pFAST were co-electroporated on one side of neural tube in ovo at embryonic day 2 (E2, HH stage 13-14). 24 h later, embryos were dissected and imaged by spinning disk confocal microscopy with the indicated imaging settings (see **Supplementary Table 13** for imaging settings). The experiment was done once. Scale bar, 10 μm.

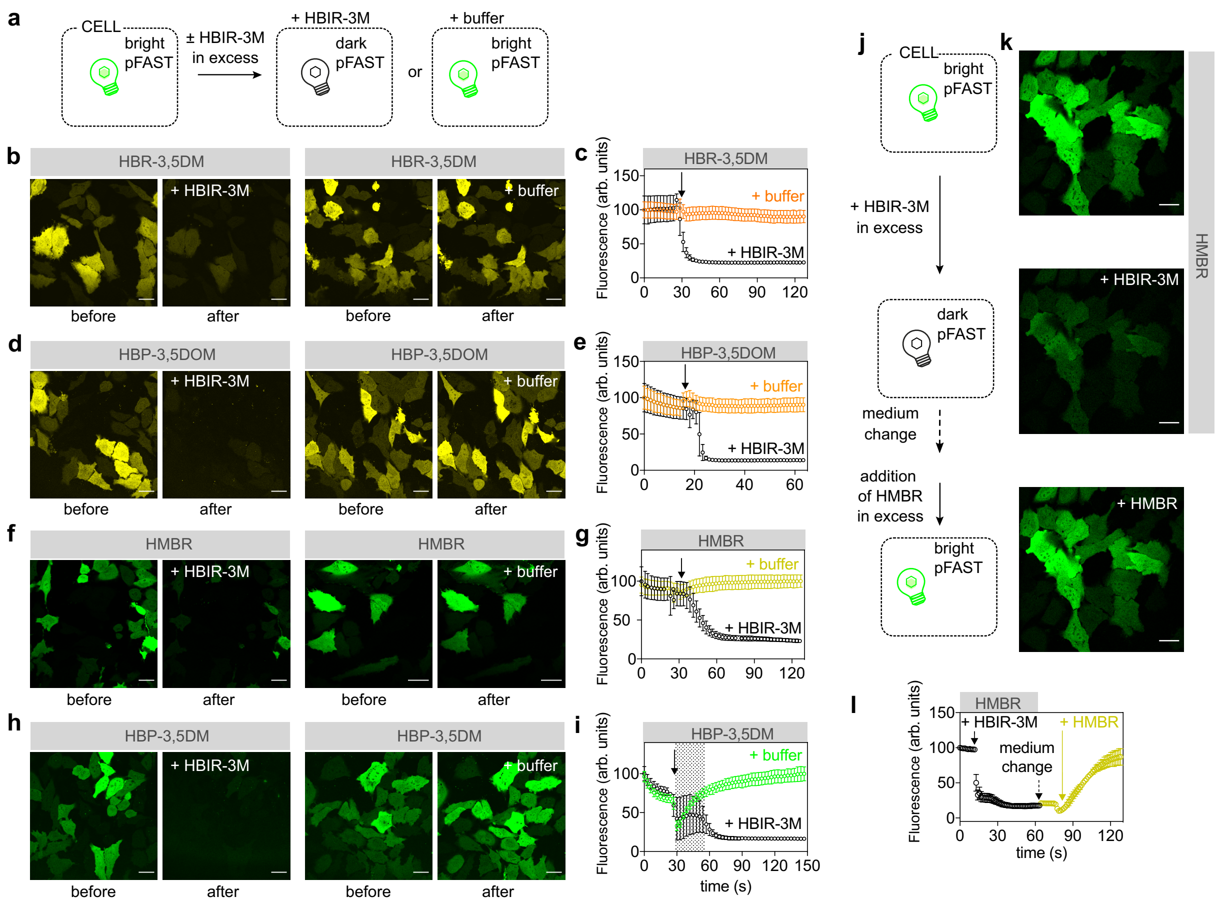

Supplementary Fig. 18. Reversible labeling of pFAST in live cells by chromophore replacement. a-i HeLa cells expressing cytoplasmic pFAST - initially labeled with **b,c** 1 µM HBR-3,5DM, **d,e** 10 µM HBP-3,5DOM, **f,g** 1 µM HMBR or **h,i** 5 µM HBP-3,5DM were treated with and without 10 µM HBIR-3M dark-competitor (keeping the concentration of fluorogenic chromophore constant) (see **Supplementary Table 13** for imaging settings). **b,d,f,h** Confocal micrographs before and after addition of the dark competitor HBIR-3M (left) or buffer (right). Scale bars, 10 µm. **c,e,g,i** Temporal evolution of fluorescence intensities upon addition of HBIR-3M or buffer. Data are represented as mean values ± SD of n = 3 cells. Addition time is indicated by the arrow (see also **Supplementary Movie 5**). Note that the artefactual jump of fluorescence on **i** (grey area) was due to a change of focus upon addition of the HBIR-3M solution or the buffer solution. **j-l** HeLa cells expressing cytoplasmic pFAST were labeled with 1 µM HMBR. Cells were then treated with 10 µM HBIR-3M dark-competitor (keeping HMBR concentration constant) until fluorescence extinction. Then medium was changed, and fresh medium with 10 µM HMBR was added. The overall experiment was followed by time-lapse fluorescence microscopy (see also **Supplementary Movie 6**). Scale bars, 10 µm. The graph on panel (l) shows the temporal evolution of the fluorescence during the experiment. Data are represented as mean values ± SD of n = 3 cells. Source data for graphs are provided as a Source Data file.

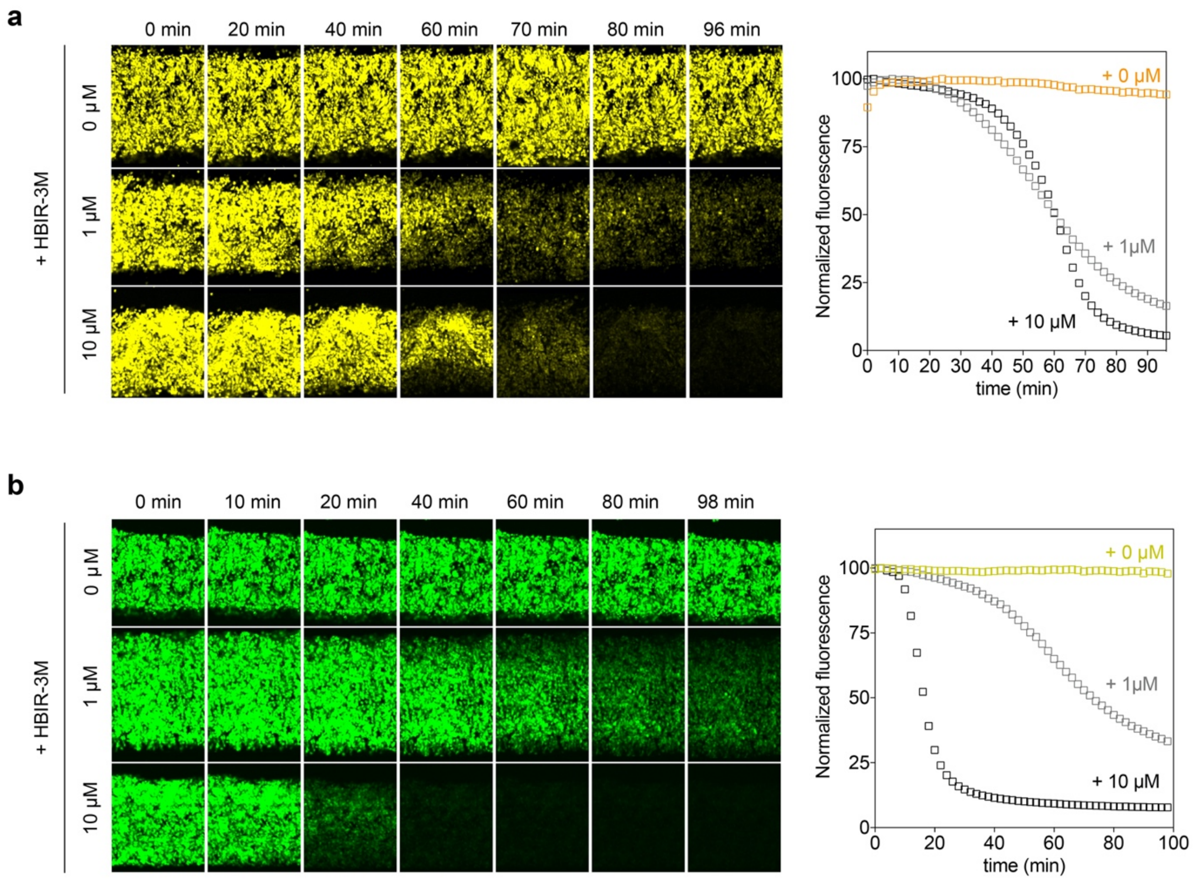

Supplementary Fig. 19. Reversible labeling of pFAST in chicken embryos by chromophore replacement. Embryos expressing H2B-pFAST in the neural tube were dissected and labeled with **a** 5 µM of HBP-3,5DOM and with **b** 1 µM of HMBR for 40 minutes (see also **Supplementary Movie 6**). The labeling solution was then removed, the samples were washed once with PBS and fresh medium supplemented with 0 µM, 1 µM and 10 µM of the dark competitor HBIR-3M was added. Time-lapse spinning-disk confocal imaging allowed to monitor the temporal evolution of the fluorescence intensity after addition of the dark competitor (see **Supplementary Table 13** for imaging settings). The graphs show the temporal evolution of the fluorescence intensities. Experiments were repeated 3 times with similar results. Source data for graphs are provided as a Source Data file.

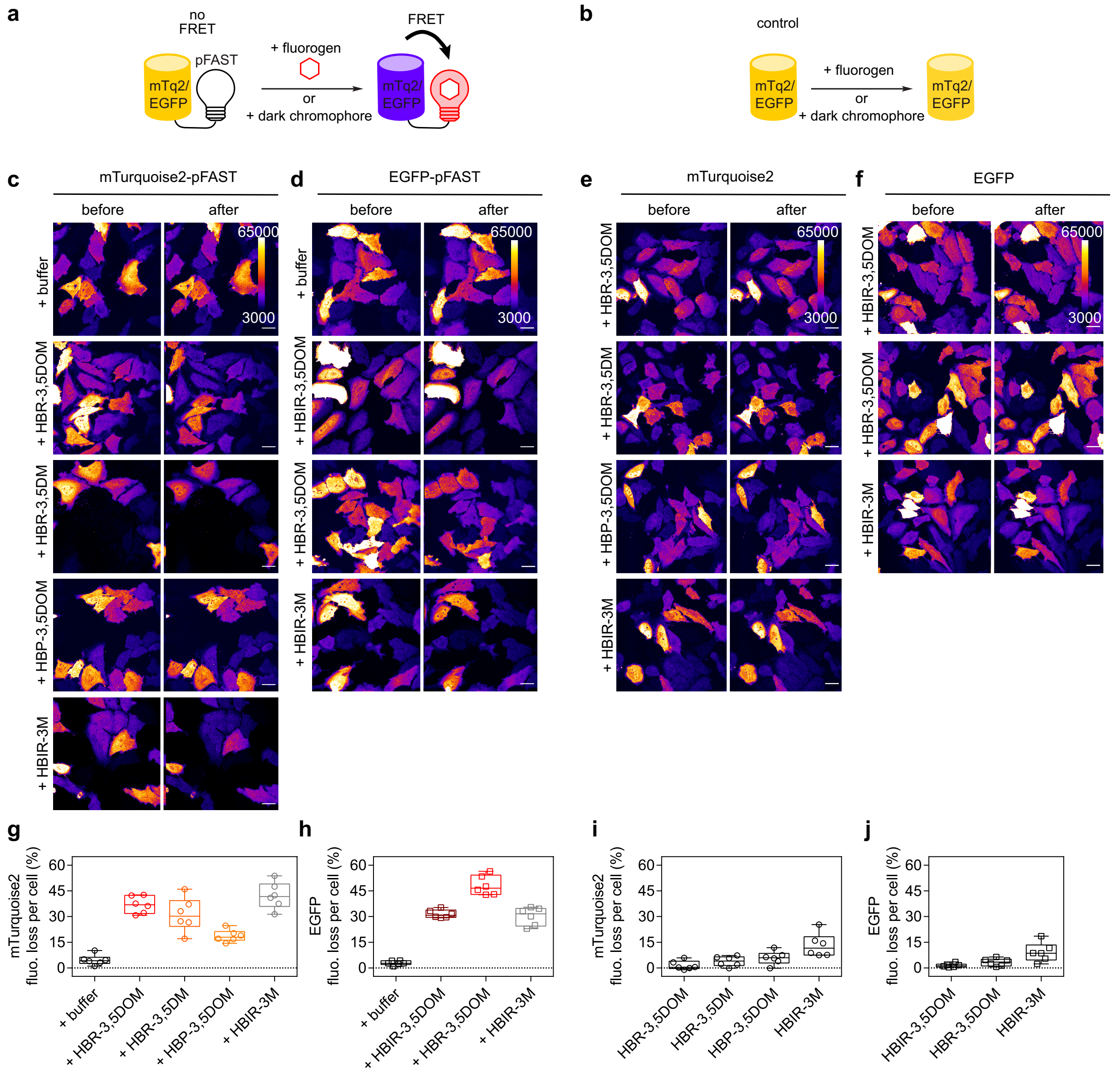

Supplementary Fig. 20. Fluorescence loss of mTurquoise2 (mTq2) or EGFP donors fused to pFAST acceptor upon addition of various chromophores in FRET experiments. a,c,d Quantification of donor fluorescence loss in live HeLa cells expressing cytoplasmic **c** mTq2-pFAST tandem before and after addition of imaging buffer, 10 μM of HBR-3,5DOM, 10 μM of HBR-3,5DM, 10 μM of HBP-3,5DOM or 10 μM of dark chromophore HBIR-3M and **d** EGFP-pFAST tandem before and after addition of imaging buffer, 10 μM of HBIR-3,5DOM, 10 μM of HBR-3,5DOM and 10 μM of dark chromophore HBIR-3M. Experiments were repeated 3 times with similar results. **b,e,f** Control experiments of fluorescence loss in live HeLa cells expressing cytoplasmic only-donor **e** mTq2 before and after addition of 10 μM of HBR-3,5DOM, 10 μM of HBR-3,5DM, 10 μM of HBP-3,5DOM or 10 μM of dark chromophore HBIR-3M and **f** EGFP before and after addition of 10 μM of HBIR-3,5DOM, 10 μM of HBR-3,5DOM or 10 μM of dark chromophore HBIR-3M (see **Supplementary Table 13** for imaging settings). Experiments were repeated 3 times with similar results. Scale bars, 30 μm. **g-j** The box plots show the **g,i** mTq2 and **h,j** EGFP fluorescence loss per cell upon addition of fluorogenic or dark chromophores (n = 6 cells). Whiskers represent the highest and lowest values. Source data for graphs are provided as a Source Data file.

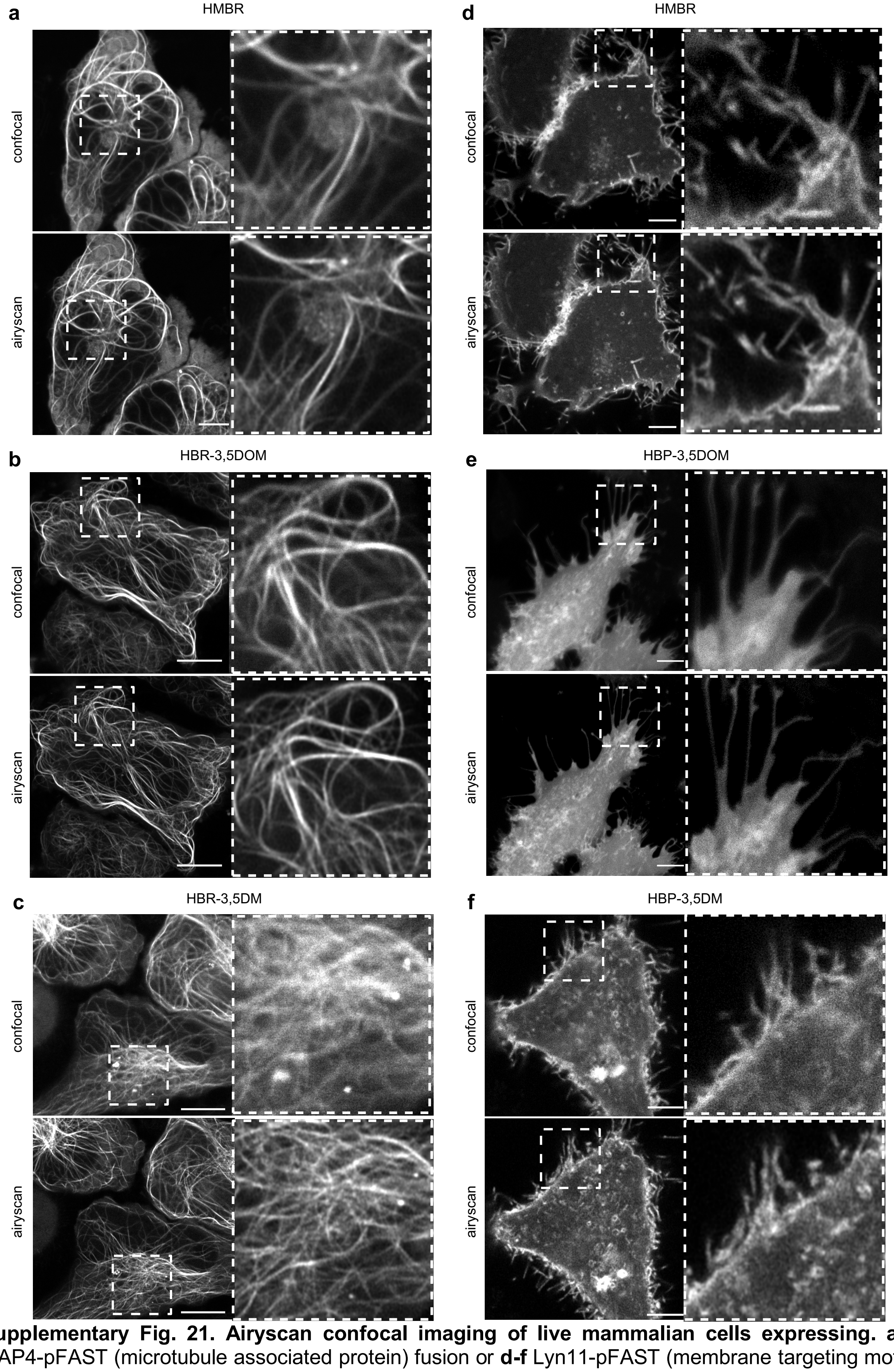

Supplementary Fig. 21. Airyscan confocal imaging of live mammalian cells expressing. a-c MAP4-pFAST (microtubule associated protein) fusion or **d-f** Lyn11-pFAST (membrane targeting motif) fusion and labeled with **a,d** 5 μM HMBR, **b** 5 μM HBR-3,5DOM, **c** 5 μM HBR-3,5DM, **e** 10 μM HBP-3,5DOM and **f** 5 μM HBP-3,5DM (see **Supplementary Table 13** for imaging settings). Experiments were repeated > 3 times with similar results. Scale bars, 10 μm.

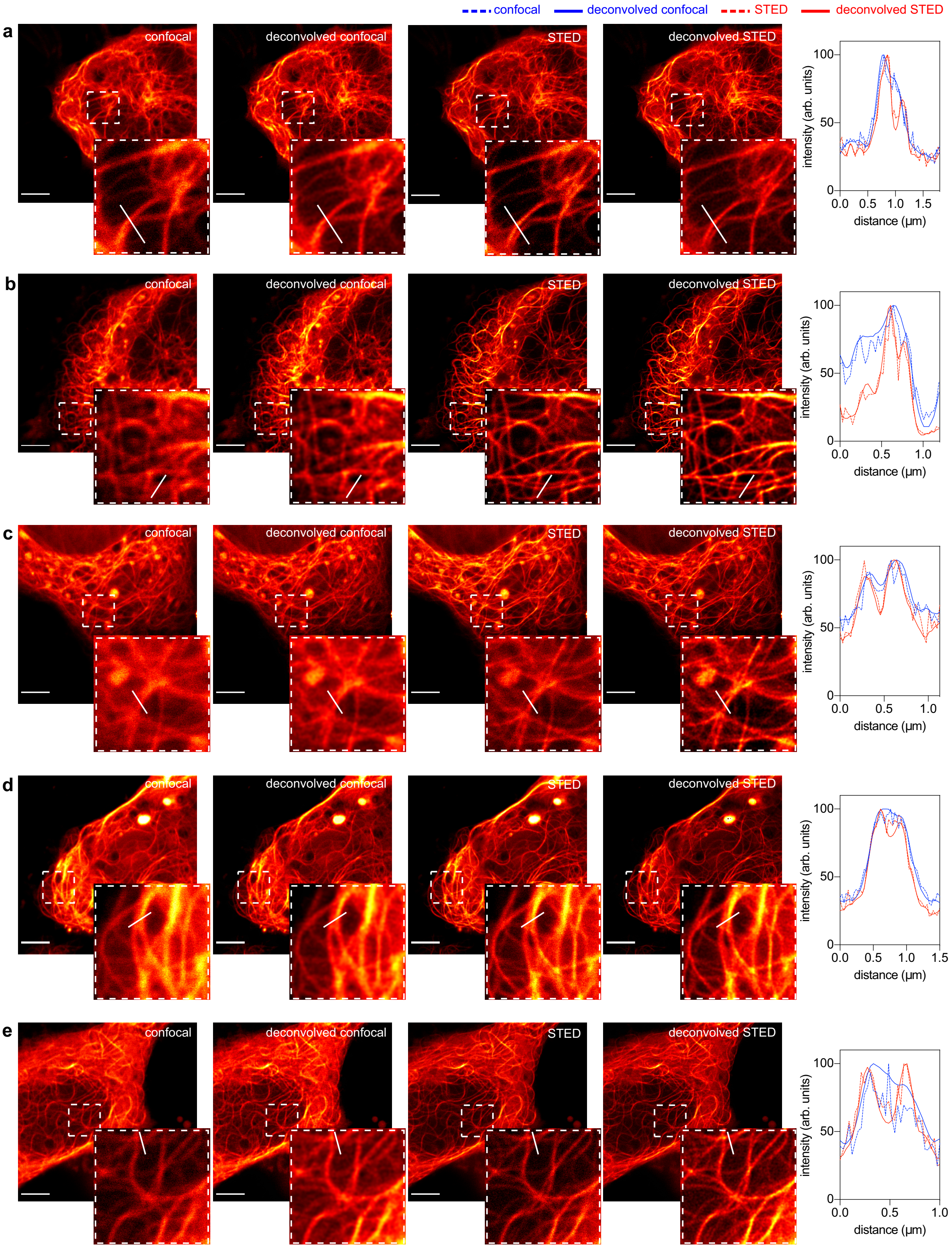

confocal
deconvolved confocal
STED
deconvolved STED
a
b
c
d
e
intensity (arb. units)
distance (µm)

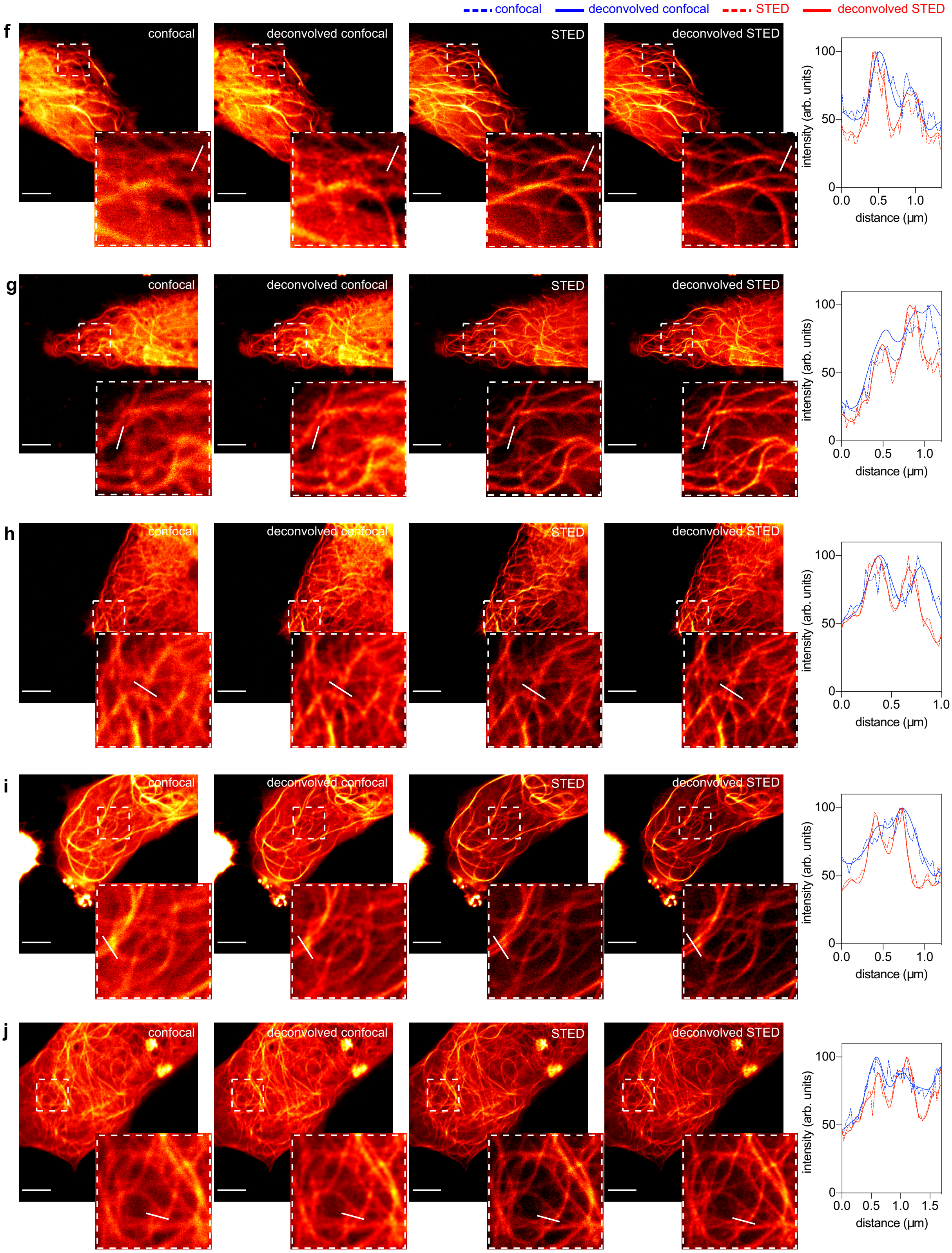

confocal
deconvolved confocal
STED
deconvolved STED
f
g
h
i
j
intensity (arb. units)
distance (μm)
100
50
0
0.0
0.5
1.0
1.5

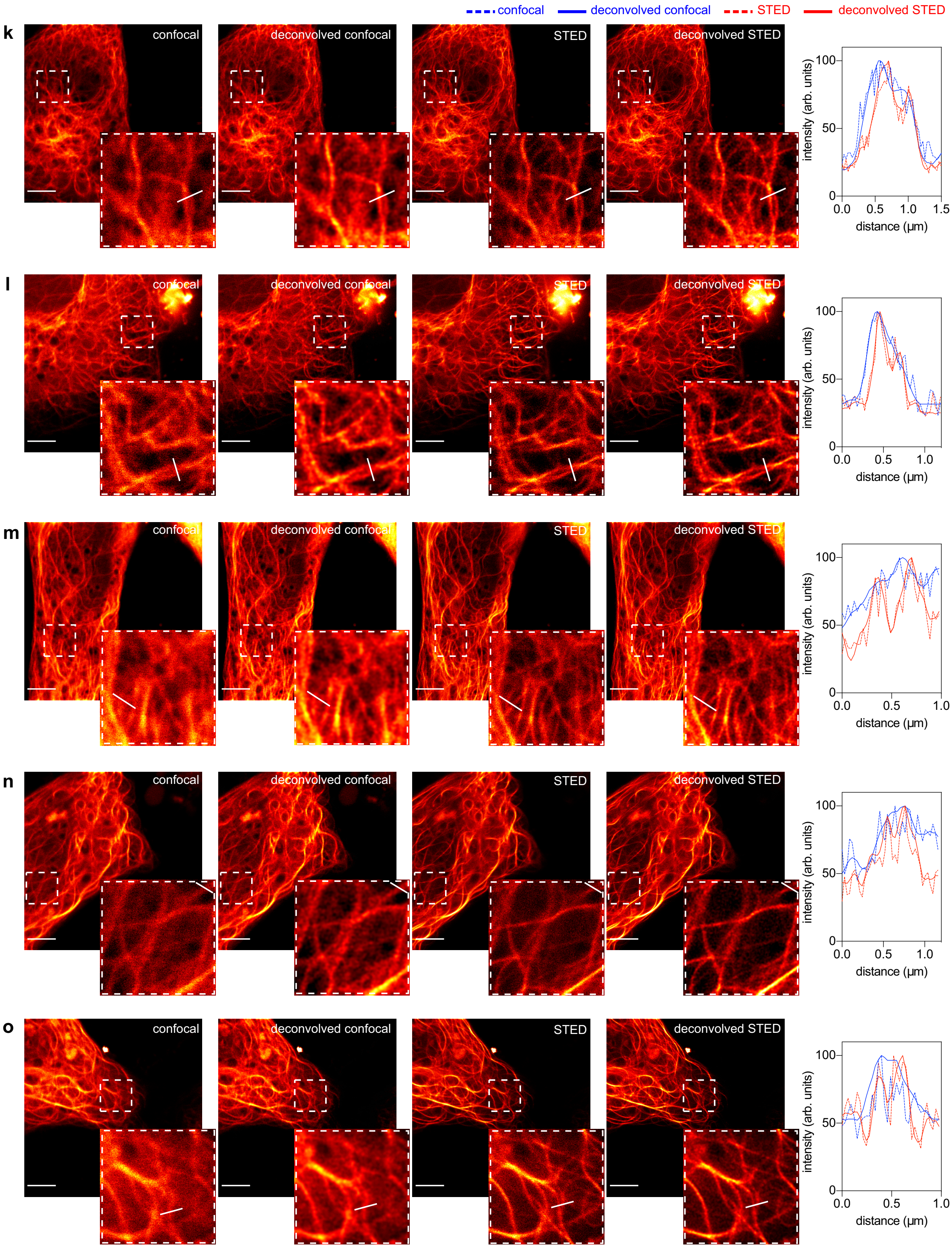

Supplementary Fig. 22. Confocal and STED micrographs of live HeLa cells expressing MAP4-pFAST. a-o Hela cells were treated with 10 μM of HBR-3,5DOM before imaging. Confocal versus STED images were acquired with the same settings. The intensity profiles enable to quantify the gain of resolution. The images were showed before (confocal and STED) and after deconvolution (deconvolved confocal and STED) (see **Supplementary Table 13** for imaging settings). Experiment was repeated 15 times with similar results. Scale bars, 5 μm. Source data for graphs are provided as a Source Data file.

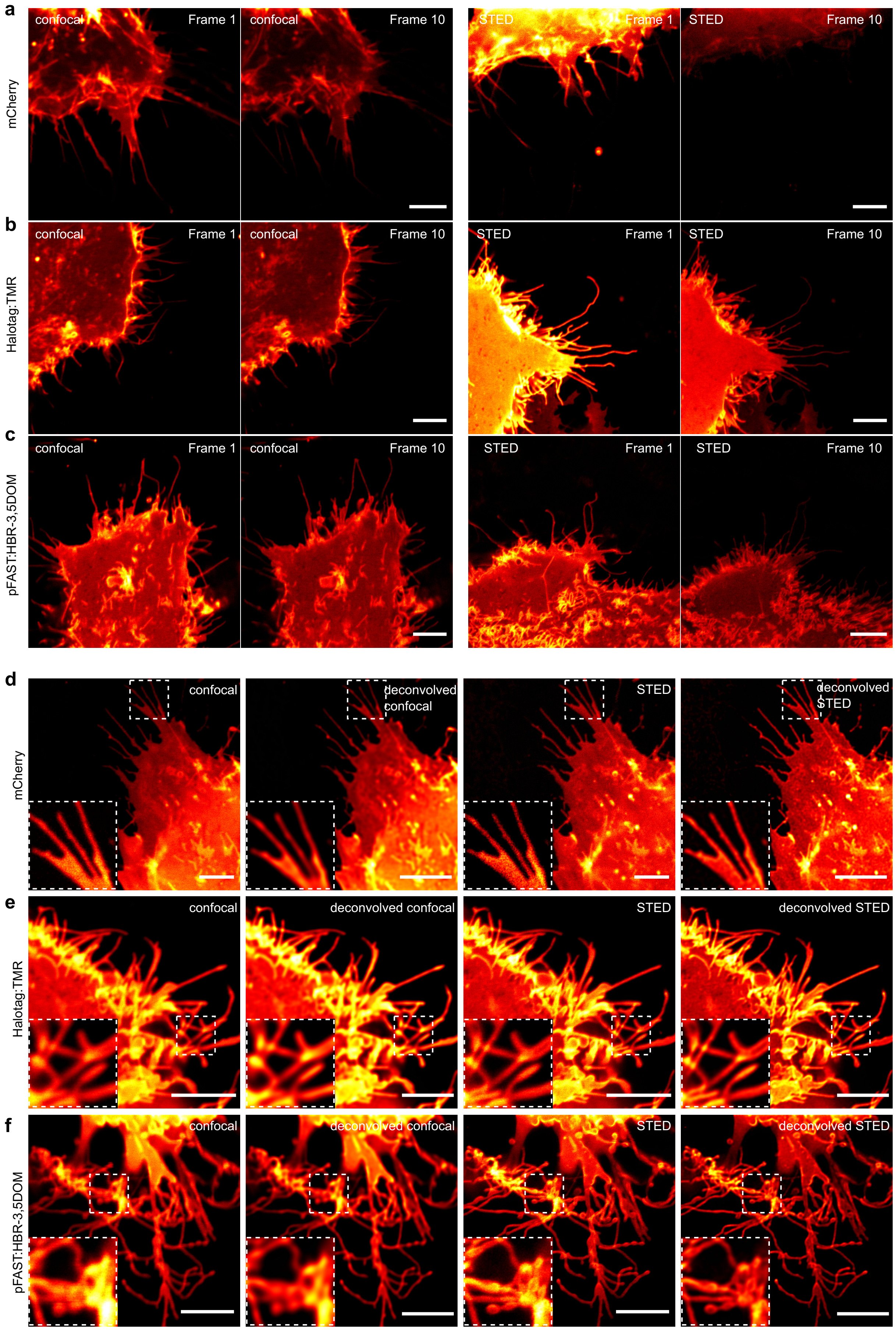
a
mCherry
confocal
Frame 1
confocal
Frame 10
STED
Frame 1
STED
Frame 10
b
Halotag:TMR
confocal
Frame 1
confocal
Frame 10
STED
Frame 1
STED
Frame 10
c
pFAST:HBR-3,5DOM
confocal
Frame 1
confocal
Frame 10
STED
Frame 1
STED
Frame 10
d
mCherry
confocal
deconvolved confocal
STED
deconvolved STED
e
Halotag:TMR
confocal
deconvolved confocal
STED
deconvolved STED
f
pFAST:HBR-3,5DOM
confocal
deconvolved confocal
STED
deconvolved STED

Supplementary Fig. 23. Comparison of pFAST, mCherry and HaloTag for STED imaging. Confocal and STED micrographs of live HeLa cells expressing **a,d** mCherry, **b,e** HaloTag or **c,f** pFAST fused to lyn11 (inner membrane-targeting motif). HaloTag was labeled with TMR ligand and pFAST with HBR-3,5DOM. **a-c** Photoresistance of **a** mCherry, **b** HaloTag or **c** pFAST when imaged by confocal microscopy or by STED. The first and tenth images of time-lapses are presented. Experiments were repeated 2 times with similar results. **d-f** Comparison of the gain of resolution when imaging **d** mCherry, **e** HaloTag or **f** pFAST by STED microscopy (vs confocal microscopy). Experiments were repeated > 8 times with similar results. Scale bars, 5 μm**.** See **Supplementary Table 13** for imaging settings

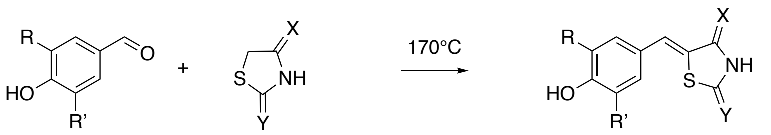

HBP-3M (R = Me, R' = H, X = O, Y = NH)
HBP-3,5DM (R = Me, R' = Me, X = O, Y = NH)
HBP-3,5DOM (R = OMe, R' = OMe, X = O, Y = NH)
HBT-3M (R = Me, R' = H, X = O, Y = O)
HBT-3,5DM (R = Me, R' = Me, X = O, Y = O)
HBT-3,5DOM (R = OMe, R' = OMe, X = O, Y = O)
HBIR-3M (R = Me, R' = H, X = S, Y = O)
HBIR-3,5DM (R = Me, R' = Me, X = S, Y = O)
HBIR-3,5DOM (R = OMe, R' = OMe, X = S, Y = O)

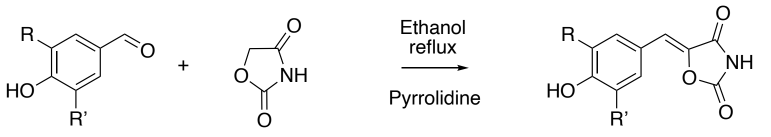

HBO-3M (R = Me, R' = H)
HBO-3,5DM (R = Me, R' = Me)

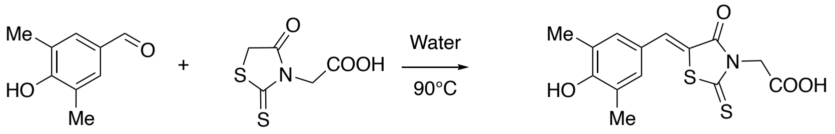

HBRAA-3,5DM

Supplementary Fig. 24. Synthetic routes for the different chromophores used in this work.

Supplementary Table 1. Properties of FAST with various chromophores in PBS pH 7.4.

Chromophore	λ_{abs} (nm)	$\Delta\lambda_{abs}$ (nm)	λ_{em} (nm)	ε ($mM^{-1}cm^{-1}$)	ϕ	Molecular brightness	K_D (μM)
HBR-3,5DOM (2)	520	114	600	39	0.31	12,000	0.97
HBR-3,5DM (2)	499	97	562	48	0.49	24,000	0.08
HMBR (2)	481	79	540	44	0.23	10,000	0.13
HBP-3,5DOM	488	121	556	31	0.29	9,000	~100
HBP-3,5DM	464	105	522	30	0.16	4,000	3.7
HBP-3M	447	87	506	21	0.044	800	6.9
HBT-3,5DOM	449	92	532	54	0.22	12,000	3.3
HBT-3,5DM	434	82	500	25	0.072	2,000	1.7
HBT-3M	415	-	480	32	0.006	200	0.24
HBO-3,5DM	409	80	481	4	0.20	700	32
HBO-3M	394	68	471	11	0.056	600	6.4

Abbreviations are as follows: λ_{abs} wavelength of maximal absorption; $\Delta\lambda_{abs} = \lambda_{abs,bound} - \lambda_{abs,unbound}$ absorption red-shift upon chromophore binding; λ_{em} wavelength of maximal emission; ε, molar absorptivity at λ_{abs} (standard error is typically 10%); ϕ, fluorescence quantum yield; molecular brightness = $\phi \times \varepsilon$; K_D thermodynamic dissociation constant.

Supplementary Table 2. Properties of oFAST with various chromophores in PBS pH 7.4

Chromophore	λ_{abs} (nm)	$\Delta\lambda_{abs}$ (nm)	λ_{em} (nm)	ε ($mM^{-1}cm^{-1}$)	ϕ	Molecular brightness	K_D (μM)
HBR-3,5DOM	519	115	600	44	0.33	14,000	0.1
HBR-3,5DM	500	98	560	53	0.38	20,000	0.01
HMBR	481	79	540	47	0.24	11,000	0.19
HBP-3,5DOM	489	122	556	28	0.30	8,000	3.0
HBP-3,5DM	465	104	519	33	0.21	7,000	0.20
HBP-3M	445	85	505	40	0.054	2,000	0.37
HBT-3,5DOM	449	92	533	68	0.21	15,000	0.30
HBT-3,5DM	435	83	501	31	0.090	3,000	0.16
HBO-3,5DM	411	82	482	6	0.30	2,000	3.0
HBO-3M	394	68	470	15	0.11	1,600	0.73

Abbreviations are as follows: λ_{abs} wavelength of maximal absorption; $\Delta\lambda_{abs} = \lambda_{abs,bound} - \lambda_{abs,unbound}$ absorption red-shift upon chromophore binding; λ_{em} wavelength of maximal emission; ε molar absorptivity at λ_{abs} (standard error is typically 10%); ϕ fluorescence quantum yield; molecular brightness = $\phi \times \varepsilon$; K_D thermodynamic dissociation constant.

Supplementary Table 3. Properties of tFAST with various chromophores in PBS pH 7.4.

Chromophore	λ_{abs} (nm)	$\Delta\lambda_{abs}$ (nm)	λ_{em} (nm)	ε ($mM^{-1}cm^{-1}$)	ϕ	Molecular brightness	K_D (μM)
HBR-3,5DOM	520	115	600	46	0.33	15,000	0.07
HBR-3,5DM	501	99	561	56	0.44	25,000	0.01
HMBR	481	79	541	52	0.24	12,000	0.19
HBP-3,5DOM	488	121	555	28	0.30	8,000	4.0
HBP-3,5DM	464	104	522	33	0.25	8,000	0.36
HBP-3M	447	87	505	39	0.075	3,000	0.56
HBT-3,5DOM	449	92	532	60	0.24	15,000	0.44
HBT-3,5DM	435	83	497	30	0.11	3,000	0.33
HBO-3,5DM	410	81	485	6	0.22	1,000	5.0
HBO-3M	393	67	471	13	0.069	1,000	0.8

Abbreviations are as follows: λ_{abs} wavelength of maximal absorption; $\Delta\lambda_{abs} = \lambda_{abs,bound} - \lambda_{abs,unbound}$ absorption red-shift upon chromophore binding; λ_{em} wavelength of maximal emission; ε molar absorptivity at λ_{abs} (standard error is typically 10%); ϕ fluorescence quantum yield; molecular brightness = $\phi \times \varepsilon$; K_D thermodynamic dissociation constant.

Supplementary Table 4. Properties of pFAST with various chromophores in PBS pH 7.4.

Chromophore	λ_{abs} (nm)	$\Delta\lambda_{abs}$ (nm)	λ_{em} (nm)	ε ($mM^{-1}cm^{-1}$)	ϕ	Molecular brightness	K_D (µM)
HBIR-3,5DOM	562	128	616	40	0.10	3,000	0.04
HBIR-3,5DM	536	106	578	25	0.015	400	0.07
HBIR-3M	514	84	567	12	0.003	50	0.005
HBR-3,5DOM	520	115	600	44	0.35	15,000	0.06
HBR-3,5DM	501	99	561	49	0.44	22,000	0.01
HMBR	481	79	542	54	0.23	13,000	0.01
HBRAA-3,5DM	524	118	578	58	0.22	12,000	1.8
HBRAA-3E	506	96	558	53	0.05	3,000	0.05
HBRAA-3M	502	96	554	64	0.08	5,000	0.23
HBP-3,5DOM	487	120	554	37	0.33	12,000	1.9
HBP-3,5DM	465	105	520	37	0.27	10,000	0.15
HBP-3M	447	87	503	35	0.10	4,000	0.22
HBT-3,5DOM	449	92	532	60	0.27	16,000	0.20
HBT-3,5DM	433	81	499	33	0.10	3,000	0.17
HBO-3,5DM	411	82	483	6	0.23	1,000	2.4
HBO-3M	392	66	473	14	0.076	1,000	0.43

Abbreviations are as follows: λ_{abs} wavelength of maximal absorption; $\Delta\lambda_{abs} = \lambda_{abs,bound} - \lambda_{abs,unbound}$ absorption red-shift upon chromophore binding; λ_{em} wavelength of maximal emission; ε, molar absorptivity at λ_{abs} (standard error is typically 10%); ϕ fluorescence quantum yield; molecular brightness = $\phi \times \varepsilon$; K_D thermodynamic dissociation constant.

Supplementary Table 5. Properties of the mutants isolated from the selection with HBO-3M compared to FAST in PBS pH 7.4.

Clone	Mutations (relative to FAST)	λ_{abs} (nm)	λ_{em} (nm)	ε ($mM^{-1}cm^{-1}$)	ϕ	Molecular brightness	K_D (µM)
FAST		394	471	11	0.056	600	6.4
A-R6.8	D65V / E93D / M109L / S117R	395	468	15	0.090	1,300	2.6
A-R6.11	V83I / M109L	394	468	15	0.074	1,100	1.4
A-R7.1	M109L	394	470	13	0.081	1,000	4.0
A-R7.2	Q41L / M109L	394	468	12	0.092	1,000	1.1
A-R7.10	V83I / T103I	394	468	12	0.088	1,000	4.4
A-R7.12	K17R / A30V / E74K / E93V	386	466	13	0.097	1,200	2.0
A-R7.14	T50S / E93Q / M95I	382	468	13	0.077	1,000	1.4

Abbreviations are as follows: λ_{abs} wavelength of maximal absorption; λ_{em} wavelength of maximal emission; ε molar absorptivity at λ_{abs} (standard error is typically 10%); ϕ fluorescence quantum yield; molecular brightness = $\phi \times \varepsilon$; K_D thermodynamic dissociation constant.

Supplementary Table 6. Properties of the mutants isolated from the selection with HBO-3,5DM compared to FAST in PBS pH 7.4.

Clone	Mutations (relative to FAST)	λ_{abs} (nm)	λ_{em} (nm)	ε ($mM^{-1}cm^{-1}$)	ϕ	Molecular brightness	K_D (µM)
FAST		409	481	4	0.20	800	32
A-R6.6	V83I / M109L	402	481	5	0.22	1,200	11
A-R6.18	H3Q / D48A / T103I / M109L	408	479	5	0.22	1,000	6.2
A-R7.1	N13I / D48E / D71N / V83G / M95V	408	483	5	0.24	1,100	5.0
A-R7.2	Q41L / M95I / M109L	406	481	6	0.25	1,500	6.1
A-R7.5	K60R / V83I / K104R / S117R	406	481	5	0.22	1,000	7.1
A-R7.7	D71N / M109L / S117R	407	482	5	0.25	1,200	4.4
A-R7.7-1	D71N / M109L / S117R + Q41L	413	480	5	0.26	1,300	2.5
A-R7.7-2	D71N / M109L / S117R + Q41L + D48E	411	480	6	0.27	1,500	2.1
A-R7.7-3	D71N / M109L / S117R + Q41L + D65V	411	483	7	0.27	1,800	2.4
A-R7.7-4 = oFAST	D71N / M109L / S117R + Q41L + V83I	411	482	6	0.30	2,000	3.0
A-R7.7-5	D71N / M109L / S117R + Q41L + M95I	411	482	5	0.26	1,400	3.0

Abbreviations are as follows: λ_{abs} wavelength of maximal absorption; λ_{em} wavelength of maximal emission; ε molar absorptivity at λ_{abs} (standard error is typically 10%); ϕ fluorescence quantum yield; molecular brightness = $\phi \times \varepsilon$; K_D thermodynamic dissociation constant.

Supplementary Table 7. Properties of the mutants isolated from the selections with HBP-3,5DM compared to FAST in PBS pH 7.4.

Clone	Mutations (relative to FAST)	λ_{abs} (nm)	λ_{em} (nm)	ε ($mM^{-1}cm^{-1}$)	ϕ	Molecular brightness	K_D (μM)
FAST		464	522	30	0.16	4,000	3.7
A-R5.3	Q41H / V83L / M95I	463	521	34	0.19	6,400	0.90
A-R5.7	Q41K / S72T / V83A / M95I	463	525	27	0.23	6,100	0.53
A-R5.12	K17N / Q41K / M95T	463	522	31	0.24	7,400	0.76
A-R5.13 =shuffling mutant 4	Q41L / S117I	463	523	32	0.21	6,600	0.71
A-R5.21 =shuffling mutant 5	V83E / S117R	463	522	33	0.20	6,400	0.69
A-R6.2	E93G / M95V / M109L	464	525	30	0.20	5,800	0.94
A-R7.1	D20N / E81Q / V83I / M109L	464	524	26	0.16	4,300	2.6
A-R7.6	G25R / A84S	464	521	32	0.23	7,100	1.2
A-R7.10	Q32K / Y76N	464	524	32	0.20	6,200	1.1
A-R5.7-1 =shuffling mutant 1	Q41K / S72T / V83A / M95I + M109L	463	519	33	0.25	8,000	0.25
A-R5.7-2	Q41K / S72T / V83A / M95I + S117I	463	521	36	0.26	9,000	0.36
A-R5.7-3	Q41K / S72T / V83A / M95I + M109L + S117I	464	521	34	0.24	8,200	0.20
A-R5.12-1 =shuffling mutant 2	K17N / Q41K / M95T + S72T + V83A	464	520	34	0.22	7,600	0.30
A-R7.6-1 =shuffling mutant 3	G25R / A84S + Q41K + M95T	465	522	34	0.26	8,700	0.98
B-R5.2	K17N / Q41K / S72T / M95T	463	521	30	0.24	7,000	0.35
B-R5.11	K17N / G25E / S72T / V83A / M95T	463	521	32	0.27	8,700	0.31
B-R5.19	Q41K / K80M / A84S / N89D / M95T / M109L	464	519	30	0.24	7,400	0.19
B-R6.1	K17N / G21E / G25R / A30V / Q41L / S72T / V83A / M95T / S117R	464	520	36	0.27	9,800	0.17
B-R6.10	Q41K / S72T / V83A / S117R	463	520	32	0.22	7,100	0.46
B-R6.15	N13S / Q41K / S72T / V83A / M95T	464	519	29	0.24	7,000	0.32
B-R6.19	K17I / G25R / Q41K / A44T / K60R / V83A / N89D / E93K / M95T /M109L / S117R	464	520	35	0.28	9,900	0.14
B-R7.3	K17N / Q41K / D65E / S72T / V83A / K106M / M109L	464	520	35	0.23	8,000	0.30
B-R6.1-1 = pFAST	K17N / G21E / G25R / A30V / Q41L / S72T / V83A / M95T / S117R + M109L	465	520	37	0.27	10,000	0.15
B-R6.1-2	K17N / G21E / G25E / A30V / Q41L / S72T / V83A / M95T / S117R	464	521	30	0.31	9,300	0.19
B-R6.1-3	K17N / G21E / G25R / A30V / Q4IK / S72T / V83A / M95T / S117R	464	521	36	0.27	9,700	0.14

Abbreviations are as follows: λ_{abs} wavelength of maximal absorption; λ_{em} wavelength of maximal emission; ε molar absorptivity at λ_{abs} (standard error is typically 10%); ϕ fluorescence quantum yield; molecular brightness = $\phi \times \varepsilon$; K_D thermodynamic dissociation constant.

Supplementary Table 8. Properties of the mutants isolated from the selection with HBT-3,5DM compared to FAST in PBS pH 7.4.

Clone	Mutations (relative to FAST)	λ_{abs} (nm)	λ_{em} (nm)	ε ($mM^{-1}cm^{-1}$)	ϕ	Molecular brightness	K_D (μM)
FAST		434	500	25	0.072	2,000	1.7
shuffling mutant 1	Q41K / S72T / V83A / M95I + M109L	432	500	28	0.090	2,500	0.26
shuffling mutant 2	K17N / Q41K / M95T + S72T + V83A	434	505	35	0.081	2,800	0.52
shuffling mutant 3	G25R / A84S + Q41K + M95T	433	501	32	0.085	2,700	0.58
shuffling mutant 4	Q41L / S117I	432	501	36	0.078	2,800	0.78
shuffling mutant 5	V83E / S117R	433	500	31	0.075	2,300	0.62
B-R6.11	A16D / Q41K / Y76H / V83A / M95T / M109L / S117R	435	497	27	0.10	2,700	0.23
B-R6.13 = tFAST	G25R/ Q41K / S72T / A84S / M95A / M109L / S117R	435	497	30	0.11	3,000	0.33
B-R6.17	A27V / Q41K/ V83A / M95T / M109L / S117I	435	499	27	0.098	2,700	0.24
B-R6.24	K17N / G21E / G25R / A30V / Q41L / S72T / V83A / M95T / S117R	434	497	29	0.094	2,700	0.26
B-R7.2	Q41K / K80R / V83A / M95T / M109L	432	496	24	0.095	2,300	0.34
B-R7.4	Q41K / M95T / S117I	432	496	26	0.095	2,500	0.53

Abbreviations are as follows: λ_{abs} wavelength of maximal absorption; λ_{em} wavelength of maximal emission; ε molar absorptivity at λ_{abs} (standard error is typically 10%); ϕ fluorescence quantum yield; molecular brightness = $\phi \times \varepsilon$; K_D thermodynamic dissociation constant.

Supplementary Table 9. Properties of the mutants isolated from the selection with HBP-3,5DOM compared to FAST in PBS pH 7.4.

Clone	Mutations (relative to FAST)	λ_{abs} (nm)	λ_{em} (nm)	ε ($mM^{-1}cm^{-1}$)	ϕ	Molecular brightness	K_D (μM)
FAST		488	556	31	0.29	9,000	100
shuffling mutant 1	Q41K / S72T / V83A / M95I + M109L	488	555	34	0.36	12,000	2.0
shuffling mutant 2	K17N / Q41K / M95T + S72T + V83A	488	556	30	0.36	11,000	2.1
shuffling mutant 3	G25R / A84S + Q41K + M95T	487	555	30	0.33	9,800	4.4
shuffling mutant 4	Q41L / S117I	485	556	31	0.33	10,000	7.2
shuffling mutant 5	V83E / S117R	488	556	32	0.30	9,500	5.9
B-R6.7	G7D / Q41K / S72T / A84S / M95I / M109L / S117R / R124L	488	555	35	0.35	12,000	4.7
B-R7.3	K17N / Q41K / A84S / M95T / M109L / S117R	487	555	33	0.37	13,000	2.8
B-R7.7	Q41K/ D48G / S72T / V83A / M95T / M109L / S117R	485	555	37	0.30	11,000	2.7
B-R7.22	G21R / G25R / Q41K / Q60E / M95T / M109L / A112G	490	557	33	0.34	12,000	5.9

Abbreviations are as follows: λ_{abs} wavelength of maximal absorption; λ_{em} wavelength of maximal emission; ε molar absorptivity at λ_{abs} (standard error is typically 10%); ϕ fluorescence quantum yield; molecular brightness = $\phi \times \varepsilon$; K_D thermodynamic dissociation constant.

Supplementary Table 10. Plasmids used in this study

Plasmid	Expression host	Open reading frame	Ref.
pCTCON-FAST	Yeast	FAST	(2)
pAG681	Yeast	shuffling mutant 1 (libraryA-R5.7-1 [HBP-3,5DM])	this study
pAG682	Yeast	shuffling mutant 2 (libraryA-R5.12-1 [HBP-3,5DM])	this study
pAG683	Yeast	shuffling mutant 3 (libraryA-R7.6-1 [HBP-3,5DM])	this study
pAG684	Yeast	shuffling mutant 4 (libraryA-R5.13 [HBP-3,5DM])	this study
pAG685	Yeast	shuffling mutant 5 (libraryA-R5.21 [HBP-3,5DM])	this study
pAG686	Yeast	pFAST	this study
pAG687	Yeast	tFAST	this study
pAG688	Yeast	oFAST	this study
pAG104	Mammalian	FAST	(2)
pAG29	Mammalian	EGFP	(2)
pAG490	Mammalian	FRB-NFAST-IRES-mTurquoise2	(3)
pAG1051	Mammalian	FRB-mTurquoise2	this study
pAG244	Mammalian	iFAST	(4)
pAG654	Mammalian	pFAST	this study
pAG655	Mammalian	tFAST	this study
pAG656	Mammalian	oFAST	this study
pAG657	Mammalian	H2B-pFAST	this study
pAG660	Mammalian	lyn11-pFAST	this study
pAG665	Mammalian	MAP4-pFAST	this study
pAG668	Mammalian	LifeAct-pFAST	this study
pAG671	Mammalian	mito-pFAST	this study
pAG897	Mammalian	pDisplay-FAST	this study
pAG876	Mammalian	pDisplay-pFAST	this study
pAG719	Mammalian	MAP4-mVenus	this study
pAG849	Mammalian	MAP4-Halotag	this study
pAG746	Mammalian	mTurquoise2-pFAST	this study
pAG862	Mammalian	EGFP-pFAST	this study
pAG744	Mammalian	mTurquoise2-AURKA-pFAST	this study
X-888	Mammalian/bird (CAG promoter)	Mito-pFAST	this study
X-858	Mammalian/bird (CAG promoter)	H2B-pFAST	this study
X-889	Mammalian/bird (CAG promoter)	H2B-FAST	this study
X-892	Mammalian/bird (CAG promoter)	H2B-mCerulean	this study
X-829	Mammalian/bird (CAG promoter)	H2B-CyoFP1	this study
pCX-H2B-EGFP	Mammalian/bird (CAG promoter)	H2B-EGFP	(5)
X-893	Mammalian/bird (CAG promoter)	H2B-EYFP	this study
pCX-H2B-mKO	Mammalian/bird (CAG promoter)	H2B-mKO	(6)
X-158	Mammalian/bird (CAG promoter)	Mb-EGFP	(7)
X-159	Mammalian/bird (CAG promoter)	Mb-mCherry	(8)
X-736	Mammalian/bird (CAG promoter)	Mb-iRFP670	(9)
pCX-PACT-mKO	Mammalian/bird (CAG promoter)	PACT-mKO	(6)

Supplementary Table 11. Clones isolated in this study (pET28 plasmids for *E. coli* expression)

Plasmid	Chromophore used for selection	Library	Clone	Mutations (relative to FAST)
pAG569	HBO-3M	A	R6.8	D65V / E93D / M109L / S117R
pAG562	HBO-3M	A	R6.11	V83I / M109L
pAG564	HBO-3M	A	R7.1	M109L
pAG565	HBO-3M	A	R7.2	Q41L / M109L
pAG566	HBO-3M	A	R7.10	V83I / T103I
pAG567	HBO-3M	A	R7.12	K17R / A30V / E74K / E93V
pAG568	HBO-3M	A	R7.14	T50S / E93Q / M95I
pAG562	HBO-3,5DM	A	R6.6	V83I / M109L
pAG563	HBO-3,5DM	A	R6.18	H3Q / D48A / T103I / M109L
pAG558	HBO-3,5DM	A	R7.1	N13I / D48E / D71N / V83G / M95V
pAG559	HBO-3,5DM	A	R7.2	Q41L / M95I / M109L
pAG560	HBO-3,5DM	A	R7.5	K60R / V83I / K104R / S117R
pAG561	HBO-3,5DM	A	R7.7	D71N / M109L / S117R
pAG795	HBO-3,5DM	A-rational design	R7.7-1	D71N / M109L / S117R + Q41L
pAG796	HBO-3,5DM	A-rational design	R7.7-2	D71N / M109L / S117R + Q41L + D48E
pAG644	HBO-3,5DM	A-rational design	R7.7-3	D71N / M109L / S117R + Q41L + D65V
pAG645	HBO-3,5DM	A-rational design	R7.7-4	D71N / M109L / S117R + Q41L + V83I
pAG797	HBO-3,5DM	A-rational design	R7.7-5	D71N / M109L / S117R + Q41L + M95I
pAG771	HBP-3,5DM	A	R5.3	Q41H / V83L / M95I
pAG772	HBP-3,5DM	A	R5.7	Q41K / S72T / V83A / M95I
pAG773	HBP-3,5DM	A	R5.12	K17N / Q41K / M95T
pAG556	HBP-3,5DM	A	R5.13	Q41L / S117I
pAG557	HBP-3,5DM	A	R5.21	V83E / S117R
pAG774	HBP-3,5DM	A	R6.2	E93G / M95V / M109L
pAG775	HBP-3,5DM	A	R7.1	D20N / E81Q / V83I / M109L
pAG776	HBP-3,5DM	A	R7.6	G25R / A84S
pAG777	HBP-3,5DM	A	R7.10	Q32K / Y76N
pAG553	HBP-3,5DM	A-rational design	R5.7-1	Q41K / S72T / V83A / M95I + M109L
pAG778	HBP-3,5DM	A-rational design	R5.7-2	Q41K / S72T / V83A / M95I + S117I
pAG779	HBP-3,5DM	A-rational design	R5.7-3	Q41K / S72T / V83A / M95I + M109L + S117I
pAG554	HBP-3,5DM	A-rational design	R5.12-1	K17N / Q41K / M95T + S72T + V83A
pAG555	HBP-3,5DM	A-rational design	R7.6-1	G25R / A84S + Q41K + M95T
pAG780	HBP-3,5DM	B	R5.2	K17N / Q41K / S72T / M95T
pAG639	HBP-3,5DM	B	R5.11	K17N / G25E / S72T / V83A / M95T
pAG781	HBP-3,5DM	B	R5.19	Q41K / K80M / A84S / N89D / M95T / M109L
pAG640	HBP-3,5DM	B	R6.1	K17N / G21E / G25R / A30V / Q41L / S72T / V83A / M95T / S117R
pAG782	HBP-3,5DM	B	R6.10	Q41K / S72T / V83A / S117R
pAG783	HBP-3,5DM	B	R6.15	N13S / Q41K / S72T / V83A / M95T
pAG642	HBP-3,5DM	B	R6.19	K17I / G25R / Q41K / A44T / K60R / V83A / N89D / E93K / M95T /M109L / S117R
pAG784	HBP-3,5DM	B	R7.3	K17N / Q41K / D65E / S72T / V83A / K106M / M109L
pAG641	HBP-3,5DM	B-rational design	R6.1-1	K17N / G21E / G25R / A30V / Q41L / S72T / V83A / M95T / S117R + M109L
pAG785	HBP-3,5DM	B-rational design	R6.1-2	K17N / G21E / G25E / A30V / Q41L / S72T / V83A / M95T / S117R
pAG786	HBP-3,5DM	B-rational design	R6.1-3	K17N / G21E / G25R / A30V / Q4IK / S72T / V83A / M95T / S117R
pAG791	HBT-3,5DM	B	R6.11	A16D / Q41K / Y76H / V83A / M95T / M109L / S117R
pAG643	HBT-3,5DM	B	R6.13	G25R/ Q41K / S72T / A84S / M95A / M109L / S117R
pAG792	HBT-3,5DM	B	R6.17	A27V / Q41K/ V83A / M95T / M109L / S117I
pAG640	HBT-3,5DM	B	R6.24	K17N / G21E / G25R / A30V / Q41L / S72T / V83A / M95T / S117R
pAG793	HBT-3,5DM	B	R7.2	Q41K / K80R / V83A / M95T / M109L
pAG794	HBT-3,5DM	B	R7.4	Q41K / M95T / S117I
pAG787	HBP-3,5DOM	B	R6.7	G7D / Q41K / S72T / A84S / M95I / M109L / S117R / R124L
pAG788	HBP-3,5DOM	B	R7.3	K17N / Q41K / A84S / M95T / M109L / S117R
pAG789	HBP-3,5DOM	B	R7.7	Q41K/ D48G / S72T / V83A / M95T / M109L / S117R
pAG790	HBP-3,5DOM	B	R7.22	G21R / G25R / Q41K / Q60E / M95T / M109L / A112G

Supplementary Table 12. Sequences of FAST and pFAST

FAST (125 amino acids, MW = 13,706 Da)
MEHVAFGSEDIENTLAKMDDGQLDGLAFGAIQLDGDGNILQYNAAEGDITGRDPKQVIGKNFFKDVAPGTDSPEFYGKFKEGVA
SGNLNTMFEWMIPTSRGPTKVKVHMKKALSGDSYWVFVKRV

DNA sequence coding for FAST (375 bp)
atggagcatgttgcctttggcagtgaggacatcgagaacactctggccaaaatggacgacggacaactggatgggttggcctttggcgcaattcagctcgatggtgacgggaatatc
ctgcagtacaatgctgctgaaggagacatcacaggcagagatcccaaacaggtgattgggaagaacttcttcaaggatgttgcacctggaacggattctcccgagttttacggcaa
attcaaggaaggcgtagcgtcagggaatctgaacaccatgttcgaatggatgataccgacaagcaggggaccaaccaaggtcaaggtgcacatgaagaaagccctttccggtg
acagctattgggtctttgtgaaacgggtg

pFAST (125 amino acids, MW = 13,884 Da)
MEHVAFGSEDIENTLANMDDEQLDRLAFGVIQLDGDGNILLYNAAEGDITGRDPKQVIGKNFFKDVAPGTDTPEFYGKFKEGAAS
GNLNTMFEWTIPTSRGPTKVKVHLKKALSGDRYWVFVKRV

DNA sequence coding for pFAST (375 bp)
atggagcatgttgcctttggcagtgaggacatcgagaacactctggccaatatggacgacgaacaactggataggttggcctttggcgtaattcagctcgatggtgacgggaatatcc
tgctgtacaatgctgctgaaggggacatcactggcagagatcccaaacaggtgattgggaagaacttcttcaaggatgttgcacctggaacggatactcccgagttttacggcaaatt
caaggaaggcgcagcgtcagggaatctgaacaccatgttcgaatggacgataccgacaagcaggggaccaaccaaggtcaaggtgcacttgaagaaagccctttccggtgac
agatattgggtctttgtgaaacgggtg

Supplementary Table 13. Imaging settings used in this study

Figure	Panel	Fluorescent reporter	Excitation settings (nm)	Emission settings (nm)	Fluorescence microscopy	Comments
Fig. 2	Panel e	oFAST:HBO-3,5DM	405	450 - 550	Confocal	
	Panel f	tFAST:HBT-3,5DM	405	450 - 550		
	Panel g	pFAST:HBP-3,5DM	488	500 - 600		
Fig. 4	Panel a	pFAST:HBO-3M	405	420 - 550	Confocal	
		pFAST:HBO-3,5DM	405	450 - 550		
		pFAST:HBT-3,5DM	405	450 - 600		
		pFAST:HBP-3M	405	450 - 600		
		pFAST:HBT-3,5DOM	488	500 - 600		
		pFAST:HBP-3,5DM	488	493 - 600		
		pFAST:HBP-3,5DOM	488	495 - 680		
		pFAST:HMBR	488	495 - 600		
		pFAST:HBRAA-3E	488	490 - 700		
		pFAST:HBRAA-3M	488	490 - 700		
		pFAST:HBRAA-3,5DM	514	550 - 700		
		pFAST:HBR-3,5DM	488	500 - 630		
		pFAST:HBR-3,5DOM	514	550 - 700		
		pFAST:HBIR-3,5DOM	561	570 - 700		
	Panel b	pFAST:HBP3,5DM	488	493 - 600		
	Panel c	pFAST:HBR-3,5DOM	520	550 - 650	Confocal	81 optical sections (z step 164 nm)
	Panel d	pFAST:HBR-3,5DOM	520	550 - 650	Confocal	Maximum intensity projection of 5 optical sections (for lyn11-pfast)
Fig. 5	Panel b	pFAST:HBR-3,5DOM<br>mKO	561	580 - 654	Spinning-disk confocal	
	Panel c	pFAST:HBR-3,5DM<br>EYFP	510/25	540/30	Widefield	LED light source
	Panel d	pFAST:HBP-3,5DOM<br>EYFP	470/24	540/30	Widefield	LED light source
	Panel e	pFAST:HMBR<br>EGFP	470/24	525/50	Widefield	LED light source
	Panel f	pFAST:HBP-3,5DOM<br>EGFP	470/24	525/50	Widefield	LED light source
	Panel g	pFAST:HBP-3M<br>mCerulean	440/20	480/40	Widefield	LED light source
	Panel h	pFAST:HBR-3,5DOM	561	580 - 654	Spinning-disk confocal	
		iRFP670	642	665 - 705		
	Panel i	pFAST:HBP-3,5DOM	488	500 - 550		
		mKO	561	580 - 654		
		pFAST:HMBR	488	500 - 550		
	Panel j	mKO	561	580 - 654		
		iRFP670	642	665 - 705		
Fig. 6	Panel b,g,h	mTurquoise2	440±10	483/35	FLIM	
	Panel d	EGFP	480±10	525/50	FLIM	
Fig. 7	Panel a-g	pFAST:HBR-3,5DOM	520	550 - 650	3D STED	775 nm pulsed depletion laser ; motorized collar 93× glycerol NA 1.3 objective; optimized pixel size(from 25 to 45 nm) and an average line acquisition of 16
	Panel i-k	pFAST:HBR-3,5DOM	520	550 - 650	3D STED	
		SiR-actin	646	658 - 770	3D STED	
Fig. 8		pFAST:HBR-3,5DOM	520	550 - 650	3D STED	775 nm pulsed depletion laser ; motorized collar 93× glycerol NA 1.3 objective; optimized pixel size(from 25 to 45 nm) and an average line acquisition of 16
Supplementary Fig. 8	Panel a,d	pFAST:HMBR	488	495 - 600	Confocal	
	Panel b	pFAST:HBR-3,5DOM	514	550 - 700		
	Panel c	pFAST:HBR-3,5DM	488	500 - 630		
	Panel e	pFAST:HBP-3,5DOM	488	495 - 680		
	Panel f	pFAST:HBP-3,5DM	488	493 - 600		
	Panel g	pFAST:HBP-3M	458	470 - 580		

	Panel h	pFAST:HBT-3,5DOM	488	495 - 600		
	Panel i	pFAST:HBT-3,5DM	458	495 - 600		
Supplementary Fig. 9	Panel a	pFAST:HBRAA-3E	488	490 - 700	Confocal	
		pFAST:HMBR	488	490 - 700		
	Panel b	pFAST:HBRAA-3M	488	490 - 700		
		pFAST:HMBR	488	490 - 700		
	Panel c	pFAST:HBRAA-3,5DM	514	530 - 700		
		pFAST:HMBR	514	530 - 700		
Supplementary Fig. 10	Panel a	pFAST:HBR-3,5DOM<br>iFAST:HBR-3,5DOM<br>FAST:HBR-3,5DOM<br>EGFP	488	550 - 700	Confocal	
	Panel b	pFAST:HBR-3,5DM<br>iFAST:HBR-3,5DM<br>FAST:HBR-3,5DM<br>EGFP	488	500 - 650	Confocal	
	Panel c	pFAST:HMBR<br>iFAST:HMBR<br>FAST:HMBR<br>EGFP	488	495 - 600	Confocal	
	Panel d	pFAST:HBR-3,5DOM<br>Halotag:TMR<br>mCherry	561	580 - 750	Confocal	
	Panel e,g,h	pFAST:HBP-3,5DOM<br>pFAST:HBP-3,5DM<br>EGFP	488	495 - 600	Confocal	
	Panel f,i,i	pFAST:HBT-3,5DOM<br>pFAST:HBP-3M<br>mTurquoise2	458	470 - 600	Confocal	
Supplementary Fig. 11		pFAST:HBP-3,5DM	488	493 - 600	Confocal	
Supplementary Fig. 12	Panel a	Halotag:TMR	561	568 - 700	Confocal	
	Panel b	mVenus	514	520 - 600		
	Panel c	pFAST:HBP-3,5DM	488	493 - 600		
Supplementary Fig. 13		pFAST:HBR-3,5DOM	520	550 - 680		
Supplementary Fig. 15	Panel a	pFAST:HBR-3,5DOM	561	580 - 654	Spinning-disk confocal	
	Panel b	pFAST:HBR-3,5DM	510/25	540/30	Widefield	LED light source
	Panel c	pFAST:HBP-3,5DOM	470/24	540/30	Widefield	LED light source
	Panel d	pFAST:HMBR	470/24	525/50	Widefield	LED light source
	Panel e	pFAST:HBP-3,5DM	470/24	525/50	Widefield	LED light source
Supplementary Fig. 17	Panel a	EGFP/CyOFP1/ pFAST:HBR-3,5DOM	488	525/50	Spinning-disk confocal	LED light source
		EGFP/CyOFP1/ pFAST:HBR-3,5DOM	488	617/73		
		EGFP/CyOFP1/ pFAST:HBR-3,5DOM	561	617/73		
	Panel b	EGFP	488	525/50		
		pFAST:HBR-3,5DOM	488	617/73		
		pFAST:HBR-3,5DOM	561	617/73		
Supplementary Fig. 18	Panel b	pFAST:HBR-3,5DM	488	500 - 630	Confocal	
	Panel d	pFAST:HBP-3,5DOM	488	495 - 680		
	Panel f,k	pFAST:HMBR	488	495 - 600		
	Panel h	pFAST:HBP-3,5DM	488	495 - 680		

Supplementary Fig. 19	Panel a	pFAST:HBP-3,5DOM	488	500 - 550	Spinning-disk confocal	
	Panel b	pFAST:HMBR	488	500 - 550		
Supplementary Fig. 20	Panel c,e	mTurquoise2	458	465 - 490	Confocal	
	Panel d,f	EGFP	488	495 - 520		
Supplementary Fig. 21	Panel a,d	pFAST:HMBR	488	495 - 550	Confocal	Equipped with a spectral detector GaAsp of 32 channels (Airyscan module)
	Panel b	pFAST:HBR-3,5DOM	561	570 - 750		
	Panel c	pFAST:HBR-3,5DM	488	525 - 620		
	Panel e	pFAST:HBP-3,5DOM	488	525 - 620		
	Panel f	pFAST:HBP-3,5DM	488	495 - 650		
Supplementary Fig. 22		pFAST:HBR-3,5DOM	488	550 - 640	Confocal	
		pFAST:HBR-3,5DOM	488	550 - 640	3D STED	775 nm pulsed depletion laser ; motorized collar 93× glycerol NA 1.3 objective; optimized pixel size(from 25 to 45 nm) and an average line acquisition of 16
Supplementary Fig. 23	Panel a,d	mCherry	585	594 - 665	3D STED	775 nm pulsed depletion laser ; motorized collar 93× glycerol NA 1.3 objective; optimized pixel size(from 25 to 45 nm) and an average line acquisition of 16 (time-lapse: 1 image / 6.883 s)
	Panel b,e	Halotag:TMR	550	560 - 650	3D STED	775 nm pulsed depletion laser ; motorized collar 93× glycerol NA 1.3 objective; optimized pixel size(from 25 to 45 nm) and an average line acquisition of 16 (time-lapse: 1 image / 6.883 s)
	Panel c,f	pFAST:HBR-3,5DOM	520	550 - 640	3D STED	775 nm pulsed depletion laser ; motorized collar 93× glycerol NA 1.3 objective; optimized pixel size(from 25 to 45 nm) and an average line acquisition of 16 (time-lapse: 1 image / 6.883 s)
Supplementary Movie 1		pFAST:HBR-3,5DOM<br>FAST:HBR-3,5DOM	561	580 - 654	Spinning-disk confocal	
		EGFP	488	500 - 550		
Supplementary Movie 2		pFAST:HBR-3,5DOM	561	580 - 654	Spinning-disk confocal	
		iRFP670	642	665 - 705		
Supplementary Movie 3		pFAST:HBP-3,5DOM	488	500 - 550	Spinning-disk confocal	
		mKO	561	580 - 654		
Supplementary Movie 4		pFAST:HMBR	488	500 - 550	Spinning-disk confocal	
		mKO	561	580 - 654		
		iRFP670	642	665 - 705		
Supplementary Movie 5		pFAST:HBR-3,5DM	488	500 - 630	Confocal	
		pFAST:HBP-3,5DOM	488	495 - 680		
		pFAST:HMBR	488	495 - 600		
		pFAST:HBP-3,5DM	488	495 - 680		
Supplementary Movie 6		pFAST:HMBR	488	495 - 620	Confocal	
Supplementary Movie 7		pFAST:HMBR	488	500 - 550	Spinning-disk confocal	

Supplementary Movie 8	pFAST:HBR-3,5DOM	561	570 - 750	Confocal	Equipped with a spectral detector GaAsp of 32 channels (Airyscan module)<br>Lyn11 : 1 image / 932 ms<br>MAP4 : 1 image /4.99 s

Supplementary References

1. H. T. S. Britton, R. A. Robinson, CXCVIII.—Universal buffer solutions and the dissociation constant of veronal. *J. Chem. Soc. Resumed*, 1456–1462 (1931).
2. M.-A. Plamont, *et al.*, Small fluorescence-activating and absorption-shifting tag for tunable protein imaging in vivo. *Proc. Natl. Acad. Sci.* **113**, 497–502 (2016).
3. A. G. Tebo, A. Gautier, A split fluorescent reporter with rapid and reversible complementation. *Nat. Commun.* **10**, 2822 (2019).
4. A. G. Tebo, F. M. Pimenta, Y. Zhang, A. Gautier, Improved Chemical-Genetic Fluorescent Markers for Live Cell Microscopy. *Biochemistry* **57**, 5648–5653 (2018).
5. A.-K. Hadjantonakis, V. E. Papaioannou, Dynamic in vivo imaging and cell tracking using a histone fluorescent protein fusion in mice. *BMC Biotechnol.* **4**, 33 (2004).
6. D. Konno, *et al.*, Neuroepithelial progenitors undergo LGN-dependent planar divisions to maintain self-renewability during mammalian neurogenesis. *Nat. Cell Biol.* **10**, 93–101 (2008).
7. S. Tozer, C. Baek, E. Fischer, R. Goiame, X. Morin, Differential Routing of Mindbomb1 via Centriolar Satellites Regulates Asymmetric Divisions of Neural Progenitors. *Neuron* **93**, 542-551.e4 (2017).
8. E. Peyre, *et al.*, A lateral belt of cortical LGN and NuMA guides mitotic spindle movements and planar division in neuroepithelial cells. *J. Cell Biol.* **193**, 141–154 (2011).
9. C. Baek, *et al.*, Mib1 prevents Notch Cis-inhibition to defer differentiation and preserve neuroepithelial integrity during neural delamination. *PLOS Biol.* **16**, e2004162 (2018).
