# Supplementary material for: Engineering of a fluorescent chemogenetic reporter with tunable color for advanced live-cell imaging
Source: Nat Commun. 2021 Nov 30;12:6989. doi: 10.1038/s41467-021-27334-0 (PMC8633346; doi:10.1038/s41467-021-27334-0)
Supplement: Supplementary file 2 — Description of Additional Supplementary Files [file 41467_2021_27334_MOESM2_ESM.pdf]

Title: Supplementary Movie 1.
Description: Labeling efficiency of pFAST and FAST with HBR-3,5DOM in dissected neural tube of chicken embryo. Plasmids encoding H2BpFAST and H2B-FAST were each electroporated in one of each side of the neural tube in ovo at embryonic day 2 (E2, HH stage 13- 14). An EGFP reporter fused to H2B was co-injected with each construct to monitor electroporation efficiency. 24 h later, embryos with homogeneous bilateral EGFP expression in the neural tube were dissected, and imaged upon successive additions of 0.1, 1 and 10 μM HBR-3,5DOM using a spinning-disk confocal microscope (see Supplementary Table 13 for imaging settings).

Title: Supplementary Movie 2.
Description: Twocolor dynamic cellular processes in dissected neural tube of chicken embryo. Plasmids encoding mito-pFAST (fused to a mitochondrial localization signal) and mbmIRFP670 (fused to a membrane localization signal) were electroporated in the neural tube in ovo, at embryonic day 2 (E2, HH stage 13-14). 24 h later, embryos were dissected, and imaged in presence of 1 μM HBR-3,5DOM using a spinning-disk confocal microscope (see Supplementary Table 13 for imaging settings). The time-lapse shows cell division. Scale bar, 10 μm.

Title: Supplementary Movie 3.
Description: Twocolor imaging in dissected neural tube of chicken embryo. Plasmids encoding H2B-pFAST (fused to histone H2B) and pact-mKO (targeting the PACT domain of pericentrin) were electroporated in the neural tube in ovo, at embryonic day 2 (E2, HH stage 13- 14). 24 h later, embryos were dissected, and imaged in presence of 5 μM HBP-3,5DOM using a spinning-disk confocal microscope (see Supplementary Table 13 for imaging settings). The time-lapse shows cell division. Scale bar, 10 μm.

Title: Supplementary Movie 4.
Description: Threecolor imaging in dissected neural tube of chicken embryo. Plasmids encoding H2B-pFAST (fused to histone H2B), pact-mKO (targeting the PACT domain of pericentrin) and mb-miRFP670 (fused to a membrane localization signal) were electroporated in the neural tube in ovo at embryonic day 2 (E2, HH stage 13-14). 24 h later, embryos were dissected, and imaged in presence of 1 μM HMBR using a spinning-disk confocal microscope. The time-lapse shows cell division (see Supplementary Table 13 for imaging settings). Scale bar, 10 μm.

Title: Supplementary Movie 5.
Description: Reversible labeling of pFAST in live mammalian cells by chromophore replacement. Time-lapse imaging of HeLa cells expressing cytoplasmic pFAST initially labeled with 1 μM HBR3,5DM, 10 μM HBP-3,5DOM, 1 μM HMBR and 5 μM HBP-3,5DM were switched off upon addition of 10 μM HBIR-3M dark-competitor (the concentration of fluorogenic chromophore was kept constant during the overall experiment). The transmitted channels allowed to visualize the cells (see Supplementary Table 13 for imaging settings). Scale bars, 30 μm.

Title: Supplementary Movie 6.
Description: Dual reversible labeling of pFAST in live mammalian cells with absorbing only-dark HBIR-3M chromophore. Time-lapse imaging of HeLa cells expressing cytoplasmic pFAST. pFAST initially labeled with 1 μM HMBR was switched off upon addition of 10 μM HBIR-3M dark-competitor (the concentration of HMBR was kept constant during the overall experiment). Then medium was changed, and fresh medium with 10 μM HMBR was added to switch on fluorescence again. The transmitted channels allowed to visualize the cells (see Supplementary Table 13 for imaging settings). Scale bars, 30 μm.

Title: Supplementary Movie 7.
Description: Reversible labeling of pFAST in dissected neural tube of chicken embryo. Plasmid encoding H2BpFAST was electroporated in the neural tube in ovo at embryonic day 2 (E2, HH stage 13-14). An mRFP reporter fused to H2B was co-injected to monitor electroporation efficiency. 24 h later, embryos with homogeneous bilateral mRFP expression in the neural tube were dissected, and initially labeled with 1 µM HMBR for 40 minutes. Time-lapse imaging allowed to monitor the fluorescence evolution after washing the embryos with PBS and addition of fresh medium supplemented with 0 µM ("wash"), 1 µM and 10 µM of the dark competitor HBIR-3M prior to imaging (see Supplementary Table 13 for imaging settings).

Title: Supplementary Movie 8.
Description: Rapid dynamics of microtubules and membranes in live cells observed with high temporal and spatial resolution by Airyscan confocal microscopy. Time-lapse imaging of HeLa cells expressing lyn11-pFAST (fused to membrane localization signal) (left) and MAP4-pFAST (microtubule associated protein) (right) and labeled with 5 µM HBR-3,5DOM allowed to visualize the rapid dynamics of membrane and microtubules with high temporal and spatial resolution by Airyscan confocal microscopy (see Supplementary Table 13 for imaging settings). Scale bars, 10 µm.
