# Supplementary material for: Engineering of a fluorescent chemogenetic reporter with tunable color for advanced live-cell imaging
Source: Nat Commun. 2021 Nov 30;12:6989. doi: 10.1038/s41467-021-27334-0 (PMC8633346; doi:10.1038/s41467-021-27334-0)
Supplement: Supplementary file 11 — Reporting Summary [file 41467_2021_27334_MOESM11_ESM.pdf]

nature research

Corresponding author(s): Arnaud Gautier

Last updated by author(s): Nov 2, 2021

Reporting Summary

Nature Research wishes to improve the reproducibility of the work that we publish. This form provides structure for consistency and transparency in reporting. For further information on Nature Research policies, see our Editorial Policies and the Editorial Policy Checklist.

Statistics

For all statistical analyses, confirm that the following items are present in the figure legend, table legend, main text, or Methods section.

n/a	Confirmed	
☐	☒	The exact sample size (*n*) for each experimental group/condition, given as a discrete number and unit of measurement
☐	☒	A statement on whether measurements were taken from distinct samples or whether the same sample was measured repeatedly
☐	☒	The statistical test(s) used AND whether they are one- or two-sided<br>*Only common tests should be described solely by name; describe more complex techniques in the Methods section.*
☒	☐	A description of all covariates tested
☒	☐	A description of any assumptions or corrections, such as tests of normality and adjustment for multiple comparisons
☐	☒	A full description of the statistical parameters including central tendency (e.g. means) or other basic estimates (e.g. regression coefficient) AND variation (e.g. standard deviation) or associated estimates of uncertainty (e.g. confidence intervals)
☐	☒	For null hypothesis testing, the test statistic (e.g. *F*, *t*, *r*) with confidence intervals, effect sizes, degrees of freedom and *P* value noted<br>*Give P values as exact values whenever suitable.*
☒	☐	For Bayesian analysis, information on the choice of priors and Markov chain Monte Carlo settings
☒	☐	For hierarchical and complex designs, identification of the appropriate level for tests and full reporting of outcomes
☒	☐	Estimates of effect sizes (e.g. Cohen's *d*, Pearson's *r*), indicating how they were calculated

Our web collection on statistics for biologists contains articles on many of the points above.

Software and code

Policy information about availability of computer code

Data collection	Data was collected and instruments were controlled using the following software packages: ZEN 2009, Zen blue and black, Leica LAS AF, Micromanager 1.4.23, Inscoper.
Data analysis	Data was analyzed using the following software packages: Prism 9, ImageJ 2.0/Fiji, Huygens, Kaluza Analysis software (Beckman Coulter). Modeling experiments were performed using Discovery studio 2019 with MODELER and CDOCKER. Solvation and minimization were performed using Adopted Basis NR algorithm.

For manuscripts utilizing custom algorithms or software that are central to the research but not yet described in published literature, software must be made available to editors and reviewers. We strongly encourage code deposition in a community repository (e.g. GitHub). See the Nature Research guidelines for submitting code & software for further information.

Data

Policy information about availability of data

All manuscripts must include a data availability statement. This statement should provide the following information, where applicable:

- Accession codes, unique identifiers, or web links for publicly available datasets
- A list of figures that have associated raw data
- A description of any restrictions on data availability

DATA AVAILABILITY
All relevant data are available from the authors. Source data of all data presented in graphs within the figures are provided with this paper. The plasmids used in this study will be available from Addgene.

April 2020

Field-specific reporting

Please select the one below that is the best fit for your research. If you are not sure, read the appropriate sections before making your selection.

- [x] Life sciences
- [ ] Behavioural & social sciences
- [ ] Ecological, evolutionary & environmental sciences

For a reference copy of the document with all sections, see nature.com/documents/nr-reporting-summary-flat.pdf

Life sciences study design

All studies must disclose on these points even when the disclosure is negative.

Sample size	No sample size calculations were performed. When relevant, the sample size (n) is provided in the corresponding figure captions. Sample sizes were chosen to support meaningful conclusions.
Data exclusions	No data were excluded
Replication	The number of replicates for each individual experiments is indicated in the figure legends. All attempts at replication were successful.
Randomization	The study does not require the allocation of samples into different experimental groups. Hence, randomization was not needed or performed.
Blinding	The study does not involve any comparisons between experimental groups, so the samples were not allocated into different experimental groups. Therefore, no blinding was needed.

Reporting for specific materials, systems and methods

We require information from authors about some types of materials, experimental systems and methods used in many studies. Here, indicate whether each material, system or method listed is relevant to your study. If you are not sure if a list item applies to your research, read the appropriate section before selecting a response.

Materials & experimental systems

n/a	Involved in the study	
☐	☒	Antibodies
☐	☒	Eukaryotic cell lines
☒	☐	Palaeontology and archaeology
☐	☒	Animals and other organisms
☒	☐	Human research participants
☒	☐	Clinical data
☒	☐	Dual use research of concern

Methods

n/a	Involved in the study	
☒	☐	ChIP-seq
☒	☐	Flow cytometry
☒	☐	MRI-based neuroimaging

Antibodies

Antibodies used	all antibodies were purchased from Invitrogen: polyclonal anti-myc chicken IgY(cat A-21281) and goat anti-chicken conjugated with Alexa 647 (cat A-21449)
Validation	25 publications report the use of the polyclonal anti-myc chicken IgY (cat A-21281) see https://www.thermofisher.com/antibody/product/Myc-Tag-Antibody-Polyclonal/A-21281

Eukaryotic cell lines

Policy information about cell lines

Cell line source(s)	HeLa (ATCC CRM-CCL2), HEK 293T (ATCC CRL-3216) and U2OS (ATCC HTB-96) cell lines were purchased from ATCC.
Authentication	None of the cell lines were authenticated after reception from ATCC.
Mycoplasma contamination	All cell lines were tested for the presence of mycoplasma. All tests were negative.
Commonly misidentified lines (See ICLAC register)	No commonly misidentified cell lines were used.

Animals and other organisms

Policy information about studies involving animals; ARRIVE guidelines recommended for reporting animal research

Laboratory animals	Chicken embryos. Embryos used in this study were between E2 (HH14) and E3 (HH14 + 24 h). The sex of the embryo was not determined.<br><br>Hippocampal neuron suspension were prepared from male and female Sprague Dawley embryonic rats (E18).
Wild animals	the study did not involve wild animals
Field-collected samples	the study did not involve samples collected on the field
Ethics oversight	Under European and French legislation on animal research, experiments on chicken embryos between E2 (HH14) and E3 (HH14 + 24 h) do not require animal ethics approval.<br><br>All experiments involving rats were performed in accordance with the directive 2010/63/EU of the European Parliament and of the Council of 22 September 2010 on the protection of animals used for scientific purposes.

Note that full information on the approval of the study protocol must also be provided in the manuscript.
